# The IgH *Eµ*-MAR regions promote UNG-dependent error-prone repair to optimize somatic hypermutation

**DOI:** 10.3389/fimmu.2023.1030813

**Published:** 2023-02-14

**Authors:** Ophélie A. Martin, Morgane Thomas, Marie Marquet, Charlotte Bruzeau, Armand Garot, Mylène Brousse, Sébastien Bender, Claire Carrion, Jee Eun Choi, Bao Q. Vuong, Patricia J. Gearhart, Robert W. Maul, Sandrine Le Noir, Eric Pinaud

**Affiliations:** ^1^ Laboratoire Contrôle de la Réponse Immune B et des Lymphoproliférations (CRIBL), Université de Limoges, CNRS Unité Mixte de Recherche 7276, Inserm Unité 1262, Limoges, France; ^2^ Centre Hospitalier Universitaire Dupuytren, Service d’Immunopathologie, Limoges, France; ^3^ The Graduate Center, The City University of New York, New York, NY, United States; ^4^ Laboratory of Molecular Biology and Immunology, National Institute on Aging, National Institutes of Health, Baltimore, MD, United States

**Keywords:** B cell, immunoglobulin gene, MARs region, somatic hypermutation (SHM), UNG

## Abstract

**Intoduction:**

Two scaffold/matrix attachment regions (5’- and 3’-*MARs_Eµ_*) flank the intronic core enhancer (c*Eµ*) within the immunoglobulin heavy chain locus (*IgH*). Besides their conservation in mice and humans, the physiological role of *MARs_Eµ_* is still unclear and their involvement in somatic hypermutation (SHM) has never been deeply evaluated.

**Methods:**

Our study analyzed SHM and its transcriptional control in a mouse model devoid of *MARs_Eµ_*, further combined to relevant models deficient for base excision repair and mismatch repair.

**Results:**

We observed an inverted substitution pattern in of *MARs_Eµ_*-deficient animals: SHM being decreased upstream from c*Eµ* and increased downstream of it. Strikingly, the SHM defect induced by *MARs_Eµ_*-deletion was accompanied by an increase of sense transcription of the IgH V region, excluding a direct transcription-coupled effect. Interestingly, by breeding to DNA repair-deficient backgrounds, we showed that the SHM defect, observed upstream from c*Eµ* in this model, was not due to a decrease in AID deamination but rather the consequence of a defect in base excision repair-associated unfaithful repair process.

**Discussion:**

Our study pointed out an unexpected “fence” function of *MARs_Eµ_* regions in limiting the error-prone repair machinery to the variable region of Ig gene loci.

## Introduction

The *IgH* locus, encoding the immunoglobulin heavy chain, is among the most complex in mammals, with multiple *cis*-regulatory elements controlling stepwise DNA accessibility to recombination and mutation through mechanisms that mainly rely on transcription (1). Current studies of the dynamic processes that regulate chromatin conformation changes and subnuclear location have renewed interest in *cis*-regulatory regions that delimit differentially regulated chromosomal domains. Among such DNA regulatory regions, nuclear Scaffold/Matrix Attachment Regions (MARs) have been implicated in the structural and functional organization of these domains. The juxtaposition of MARs to intronic enhancer elements in both *IgH* and *IgL* loci and their conservation in humans, mice and rabbits (2) suggest that such regions serve physiological functions. They participate in the regulation of gene expression notably by increasing enhancer function and facilitating their action over large distances. Several proteins found to bind MARs are expressed ubiquitously or in a tissue-specific manner, respectively defining constitutive or facultative MARs (3). Once attached to the nuclear matrix in a tissue specific fashion, facultative MARs could form topological barriers that could isolate or fasten chromatin regions (3). Such barriers could induce DNA torsional strain with positive and negative DNA supercoiling, respectively, upstream and downstream from the RNA pol II-induced transcription bubble (4). The supercoils are then released by the action of dedicated topoisomerases (5).

The *IgH Eµ* enhancer region is a combination of both the core *Eµ* (*cEµ*) enhancer element (220 bp) and two 310–350-bp flanking *MARs* (*MARs_Eµ_*) that were first defined by *in vitro* matrix-binding assays (6). This region, especially *cEµ*, controls early VDJ recombination events (7, 8) and is also involved in Ig *µ* chain expression in pre-B cells (9). However, its role in SHM remains unclear. An elegant model of deletion in the endogenous *Eµ* region of hybridoma cells, enforced for human AID expression, suggested the requirement of *cEµ* and a substantial function of *MARs_Eµ_* for SHM (10). Similarly, when added to transgenes, *cEµ* and its flanking *MARs* contribute to Ig *µ* chain expression and high levels of SHM (11–16). In contrast, knock out (KO) models underlined the complexity of its physiological regulation. In a mouse model carrying the pre-rearranged VB1-8i region, *Eµ* deletion still resulted in a high level of SHM in Peyer’s patch germinal center (GC) B cells, arguing for a non-essential role of the enhancer (17). More clearly, deletion of *cEµ* in the mouse germline did not reduce SHM frequency but only slightly increased the proportion of unmutated alleles; this minor effect was likely due to the reduced inflow of peripheral and, consequently, GC B cells in this model (7). Strikingly, the role of *MARs_Eµ_* was also elusive and somewhat controversial. Whereas their endogenous deletion, analysed in mouse chimeras by the RAG-2 complementation assay, demonstrated that *MARs_Eµ_* are dispensable for VDJ recombination and IgH expression (18), the ambiguous function of *MARs_Eµ_* was sustained by the discrepancy between their ability to either bind negative regulatory factors (19, 20), improve *cEµ* enhancer efficiency (21), or substitute for *cEµ* to maintain IgH expression (22). At the κ light chain locus (*Igκ*), the intronic enhancer *Eiκ* region also contains an upstream *MAR.* The implication of *MAR_Eiκ_* as an enhancer of SHM was first suggested in transgenic studies (23) and then tested in a KO mouse model that accumulated premature light chain rearrangements with a mild SHM defect (24), an effect comparable to one observed at *IgH* locus in hybridoma cells devoid of *MARs_Eµ_* (10). At that time, while these studies instigated a variety of hypotheses accounting for MARs in modulating SHM (25), these were contradicted by a study comparing *3’Eκ*-and *MAR*_Eiκ_- function in mouse KO models (26).

To address the controversy over the role of the scaffold in SHM, we generated a mouse model carrying a germline deletion of *MARs_Eµ_* and bred it into DNA repair-deficient backgrounds. In our models devoid of *MARs_Eµ_* and their *wt* counterparts, we proceeded to side by side comparison of total SHM, transcription patterns, AID targeting and error prone repair events leading to SHM, in regions located upstream and downstream from the intronic enhancer. Our study showed that the absence of *MARs_Eµ_* allows some of the error-prone repair machinery to get access to the region downstream from the *Eµ* enhancer. We propose that *MARs_Eµ_* act as physiological barriers for error-prone repair in activated B cells. As a rational hypothesis, our study suggests that the conservation of nuclear matrix attachment regions in Ig genes serves to optimize SHM events upstream from the intronic enhancer region.

## Results

### Normal B cell development and Ig production in the absence of *MARs_Eµ_*

**Figure 1:**
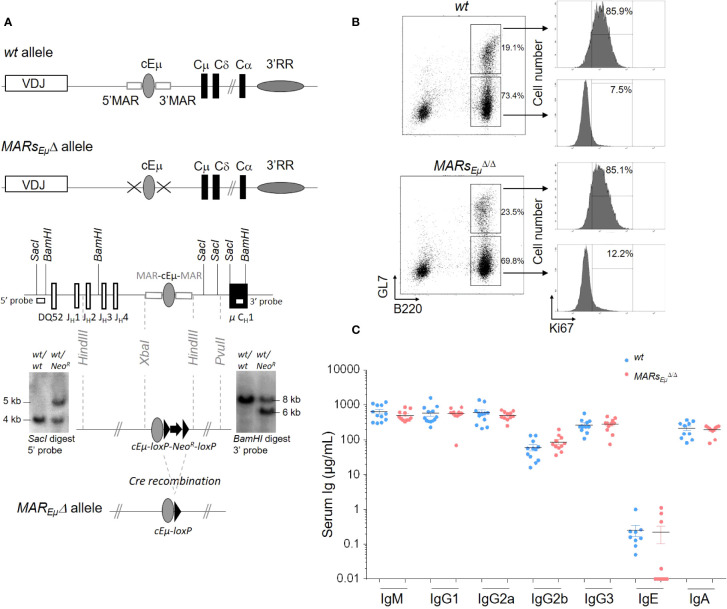
*MARs_Eµ_* deletion supports efficient *in vivo* Ig isotype production and GC B cell development. **(A)** Schematic representation of *wt* and *MARs_Eµ_* ^Δ^ alleles (top). Targeting construct and Southern blot performed on recombinant ES cells with *Neo^R^* insertion. Hybridization with the 5′ probe detected 4 kbp and 5 kbp *SacI* genomic fragments respectively for *wt* and recombined alleles. Hybridization with the 3′ probe detected 8 kbp and 6 kbp *BamHI* genomic fragments respectively for *wt* and recombined alleles. *MARs_Eµ_* ^Δ^ allele preserved the *cEµ* enhancer after Cre-recombination. **(B)** Comparison of Peyer’s patch B cells subsets from *wt* and *MARs_Eµ_* ^Δ/Δ^ animals by flow cytometry: dot plots showed percentage of naïve (B220^+^/GL7^-^) and GC (B220^+^/GL7^+^) B cells (left panels) and, for each subset, the percentage of dividing cells (Ki67^+^) was indicated on cell count histogram plots (right panels). Experiments were performed twice with a minimum of 3 mice per group. **(C)** Immunoglobulin isotype secretion in sera from *wt* and *MARs_Eµ_* ^Δ/Δ^ mice determined by ELISA (n=9 to 12 mice, mean ± SEM).

We generated a mouse mutant line carrying an endogenous deletion of both the 5’ and 3’ *IgH* matrix attachment regions that flank the *J_H_-C_H_* intronic *cEµ* enhancer. Although generated with slightly different targeting vector backbone and homology arms, the resulting *IgH* allele, so-called *MARs_Eµ_* ^Δ^ (**Figure 1A**), is similar to that generated by Sakai et *al*. (18). Bone marrow subsets of B cell precursors were analysed in *wt* and homozygous *MARs_Eµ_* ^Δ/Δ^ deficient mice. When compared to age-matched *wt* animals, *MARs_Eµ_* ^Δ/Δ^ mice exhibited normal proportions and numbers of pre-proB, pro-B and pre-B cell precursors (**Supplementary Table 1**). Unlike endogenous deletion of the entire *Eµ* region (9), *MARs_Eµ_* deletion did not modify Ig *µ* heavy chain expression in early B lineage cells since proportions of IgM-expressing bone marrow B cell populations (immature, transitional and mature recirculating B cell subsets) were comparable to those of *wt* (**Supplementary Table S1** and **Supplementary Figure S1A**). Mature B cell subsets were also similar to *wt* in the spleen and peritoneal cavity of homozygous *MARs_Eµ_* ^Δ/Δ^ mutants (**Supplementary Table S1** and **Supplementary Figures S1B, C**). In agreement with the normal inflow of mature B cells in *MARs_Eµ_* ^Δ/Δ^ animals, Peyer’s patches were efficiently colonized by naive and GC B cells. Numbers and proportions of GC B cells were even significantly increased in homozygous mutants (**Figure 1B** left panels and **Supplementary Table S1**). The similar proportion of proliferating KI67^+^ GC B cells in *wt* and *MARs_Eµ_* ^Δ/Δ^ mice implied that this increase was not due to over-proliferation of Peyer’s patch B cells (**Figure 1B** right panels). Finally, levels of serum Ig isotypes were unaffected in *MARs_Eµ_* ^Δ/Δ^ animals (**Figure 1C**). This normal B cell homeostasis in homozygous mutants confirmed that *MARs_Eµ_* are dispensable for B cell ontogeny and antibody production. This statement is in agreement with previous studies of an analogous MAR region in the *Igκ* locus (24).

### *MARs_Eµ_* deletion inverts SHM distribution on both sides of the *Eµ* enhancer region

**Figure 2:**
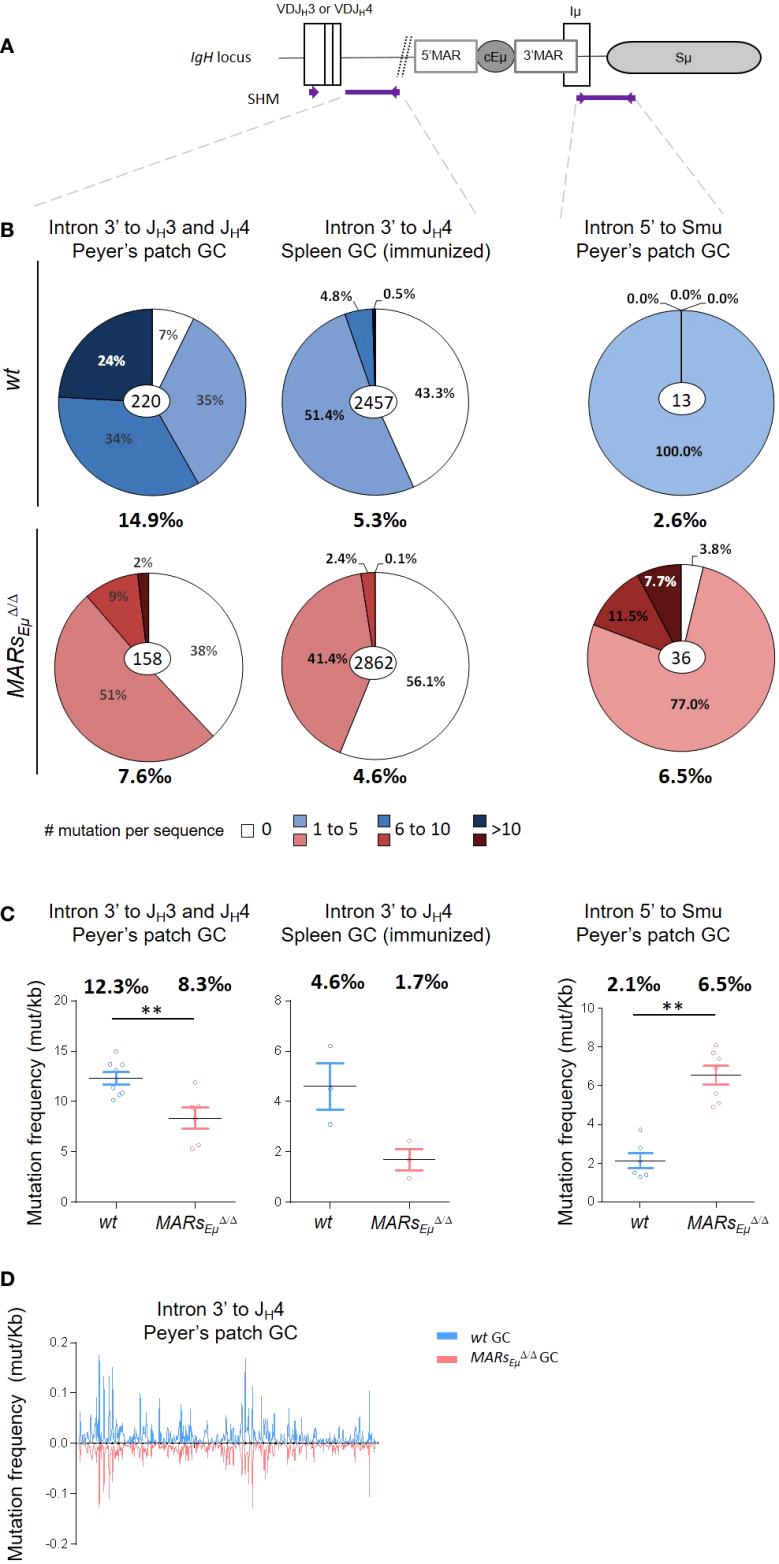
*MARs_Eµ_* deletion impairs the overall SHM frequency and distribution within the *IgH J-C* intronic region. **(A)** Location of *IgH* regions (thick purple lines) tested for SHM, arrows represent primers used for PCR amplification. **(B)** Pie charts represent distribution of mutated sequences (proportional to the area in each slice, data obtained by Sanger and GS Junior sequencing method) quantified in *wt* and *MARs_Eµ_* ^Δ/Δ^ mice in individually recombined *IgH* alleles. For each genotype number of individual clones is indicated in the center (after removal of clonally related sequences based on VDJ junction) and overall mutation frequencies (mutation per 1000 bp in mutated clones) are indicated below. Left: SHM downstream from *J_H_3* and *J_H_4* segments in Peyer’s patch sorted GC B cells, data obtained after cloning and sequencing by classical Sanger method. Middle: SHM downstream from *J_H_4* segments in spleen GC B cells sorted from SRBC-immunized mice, data obtained by NGS (GS Junior). Right: SHM downstream from *cEµ* region from Peyer’s patch GC sorted B cells, data obtained by classical Sanger method. **(C)** Graphical representation of SHM frequency in *wt* and *MARs_Eµ_* ^Δ/Δ^ mice, quantified by NGS (Ion Proton) submitted to DeMinEr filtering, a pipeline that identifies substitution frequency at each nucleotide based on an *Aicda^Δ/Δ^* control sample (28). Since no indication in sequence distribution is available using this method, data were represented as scattered plots, each point refers to a mutation frequency from one individual mice, mean mutation frequencies are indicated above. p-value was determined with two tailed Mann Whitney test; significant differences are indicated by: **P < 0.01 and error bars represent SEM of two independent experiments. **(D)** Mutation distribution along the *J_H_4* intron in *wt* (top) and in *MARs_Eµ_* ^Δ/Δ^ (bottom).

To assess whether *MARs_Eµ_* deletion could affect *IgH* somatic hypermutation, we first quantified mutations within the 500-bp regions downstream from the variable exons rearranged to *J_H_3* and *J_H_4* segments (**Figure 2A**) in Peyer’s patch GC B cells sorted from *wt* and *MARs_Eµ_* ^Δ/Δ^ (animals overall data reported in **Figure 2** left, data from individual animals reported in **Supplementary Figure S2** and **Supplementary Tables S2A, B**). For this we used two complementary sequencing methods: the first one, based on classical Sanger approach and GS junior technology, allowed to discriminate and exclude unmutated and clonally related sequences from the calculation of SHM frequency, as initially described (27). The second method used Ion proton deep sequencing coupled to DeMinEr filtering. Since using DNA templates including non-mutated alleles, this second approach underestimated the SHM frequency; but since including AID-deficient control samples as a reference, the method provided highly reproducible and reliable quantification of SHM in a DNA sample extracted from GC B cells (28). Interestingly, by using Sanger approach, *MARs_Eµ_* ^Δ/Δ^ GC B cells displayed significant differences in the distribution of mutations: an increased proportion of unmutated sequences (less than 10% in *wt* compared to 38% in *MARs_Eµ_* ^Δ/Δ^) (from 30.6 to 45.5%, overall data collected from several mice, data from independent mice in **Supplementary Figure S2A**) Another effect of *MARs_Eµ_* deletion on *IgH* SHM targeting was the strong decrease in highly mutated sequences (>10bp per sequence). In *wt*, the proportion of highly mutated alleles reached ~24% (from 18.9 to 28.3%) while in mutants they were barely present ( ∼2%) (**Figure 2B** left and **Supplementary Fig S2A**). When comparing only the mutated sequences, mutation frequency was decreased at least by two fold in *MARs_Eµ_* ^Δ/Δ^ mutants, with 7.6 mutations per 1000 bp (in average) compared to 14.9 in *wt* (in average) (**Figure 2B** left and **Supplementary Figure S2A**). By using next generation sequencing (NGS), the decreased SHM frequency was also highly significant (**Figure 2C**, 12.3‰ *vs* 8.3‰, p=0.008, individual mice in **Supplementary Table S2A**).

To monitor SHM upon antigen challenge, we analyzed mutations by Sanger and NGS in a large number of GC B cells sorted from spleen of SRBC-immunized *MARs_Eµ_* ^Δ/Δ^ and *wt* mice. In parallel; we verified on spleen frozen section that efficient GC formation was indeed comparable between *MARs_Eµ_* ^Δ/Δ^ and *wt* mice (**Supplementary Figure S2B**). In the intronic region downstream from the *J_H_4* segment, SHM frequency dropped from 5.3 (Sanger method, excluding unmutated clones) or 4.6 (NGS bulk method) mutations per 1000bp in *wt* cells to respectively 4.6 or 1.7 in *MARs_Eµ_* ^Δ/Δ^ cells (**Figure 2B** middle, and **2C**, **Supplementary FigureS2C**, **Supplementary Table S2B**). Similarly to what was observed in spontaneous GC B cells from Peyer’s patches, NGS assays performed on few immunized animals also showed that SHM was decreased upon *MARs_Eµ_*-deletion only eight days after antigen challenge (**Figure 2C** middle). Immunization assays also showed an increased proportion of unmutated or poorly mutated sequences in *MARs_Eµ_*-deficient B cells (**Figure 2B** and **Supplementary Figure S2C**). This confirmed that the intronic region was less efficiently targeted by SHM in *MARs_Eµ_* deficient mice. An identical SHM defect was also observed in mice harbouring deletion of the entire *Eµ* region (core enhancer and flanking MARs; (9)) (**Supplementary Figure S2A** top right); this data indicated that the SHM failure was the only consequence of *MARs* deletion. This hypothesis is completely consistent with a previous study showing that SHM efficiency was not affected by the endogenous deletion of the *cEµ* enhancer alone (7). The comparison between those three models is certainly relevant since all knock outs were created in murine germlines with similar mixed genetic backgrounds.

To reinforce the unbiased SHM evaluation within *J_H_* introns, we sought to evaluate the impact of *MARs_Eµ_* deletion on acquired diversity of the VDJ exon by quantifying SHM events taking place in the out of frame (passenger) IgH alleles. This parallel unbiased analysis was made possible in a region physiologically highly targeted by SHM (overlapping parts of the FR3 and CDR3 domains) thanks to the IMGT/HighV-QUEST reference tool (29). Although focused on a restricted region of out of frame VDJ exons, this study performed on GC cells sorted from Peyer’s patches still highlighted a decreased SHM frequency in the *MARs_Eµ_* ^Δ/Δ^ model (∼27‰ in mutants compared to ∼32‰ in *wt*, **Supplementary Table S3**). This strongly suggests that the SHM defect observed within *J_H_* introns in the absence of *MARs_Eµ_* extends to their respective upstream VDJ exons.

Since our *MARs_Eµ_* deletion includes the 3’*HinfI-XbaI* genomic region that contains transcription start sites and part of the *Iµ* exon (6, 30), we also quantified SHM immediately downstream from this exon in a 629bp region described as mutated in GC B cells (**Figure 2A**) (31, 32). Unlike the intronic regions downstream from the rearranged *VDJ* exon, the overall mutation frequency downstream from *Iµ* was strongly increased in GC B cells devoid of *MARs_Eµ_* region and reached 6.5 (in average) mutations per 1000 bp compared to 2.6 (in average) in *wt* cells (**Figure 2B** right). This suggests that the region downstream from the *cEµ* was more efficiently targeted in the absence of its *MARs.* This was supported by the very low proportion of unmutated sequences in wt (**Figure 2B** right and **Supplementary Figure S3A**) and the increased proportion of highly mutated sequences in *MARs_Eµ_*-deficient GC B cells (**Figure 2B** right and **Supplementary Figure S3A**). This data was efficiently confirmed by NGS analysis that estimated that mutation frequency was increased by 3.5 fold in the *MARs_Eµ_*-deficient GC B compared to *wt* mice (2.1‰ *vs* 6.5‰) (**Figure 2C** right and **Supplementary Table S2C**). In our mouse model, 3’MARs deletion brought *Sµ* region closer to the *cEµ* enhancer element. Given this, we also calculated SHM frequency in a 473bp region located at the same distance from the *cEµ* element in *wt* and *MARs_Eµ_*-deficient mice. Similarly to what was reported in **Figure 2**, we also observed, at the same distance of *cEµ*, an increase in SHM frequency upon *MARs_Eµ_*-deletion (**Supplementary Figure S3B**).

Analysis of mutation distribution in *wt* and in *MARs_Eµ_* ^Δ/Δ^ mice did not show any difference between models (**Figure 2D**), indicating that, while affecting SHM efficiency, the absence of *MARs_Eµ_* region did not influence DNA sequence hotspot or preferences for SHM within the *J_H_4* intron.

Importantly, our mouse model clearly assigns a specific function for endogenous *MARs_Eµ_* on SHM at the *IgH* locus, in accord with the requirement of similar regions for efficient SHM previously pointed out in the endogenous *Igκ* Kappa light chain locus. This pioneer study, describing the specific deletion of a 420bp MAR region upstream from the intronic Kappa enhancer (*Eiκ*), highlighted a modest decrease in SHM by quantifying mutations downstream from the *J_κ_5* segment in GC B cells from Peyer’s patches (24). While our study suggests that *MARs_Eµ_* optimizes SHM upstream from the *cEµ* enhancer; the presence of such regulatory regions does not prevent the SHM machinery to get access to downstream regions as reported in a recent study (33). This hypothesis is mostly supported by the increased SHM frequency downstream from the *cEµ* enhancer in the absence of *MARs_Eµ_*, a finding consistent with previous works describing increased *Sµ* internal deletions in hybridomas devoid of MARs regions (34). We could speculate that one physiological function of *MARs_Eµ_* regions in GC B cells is to tightly isolate the *VDJ* transcription unit by, at least temporarily, attaching the *Eµ* region to the nuclear matrix. Such a “locked” target conformation could provide an optimal environment for somatic mutations by trapping the transcription machinery and its co-factors including AID and error-prone repair factors. This topological barrier could, at the same time, partially protect downstream constant regions from SHM; although this configuration should be brief since regions downstream from *Eµ* are also efficiently targeted by AID in GC B cells (35).

### *MARs_Eµ_* deletion modifies transcription patterns on both sides of the *Eµ* enhancer region

**Figure 3:**
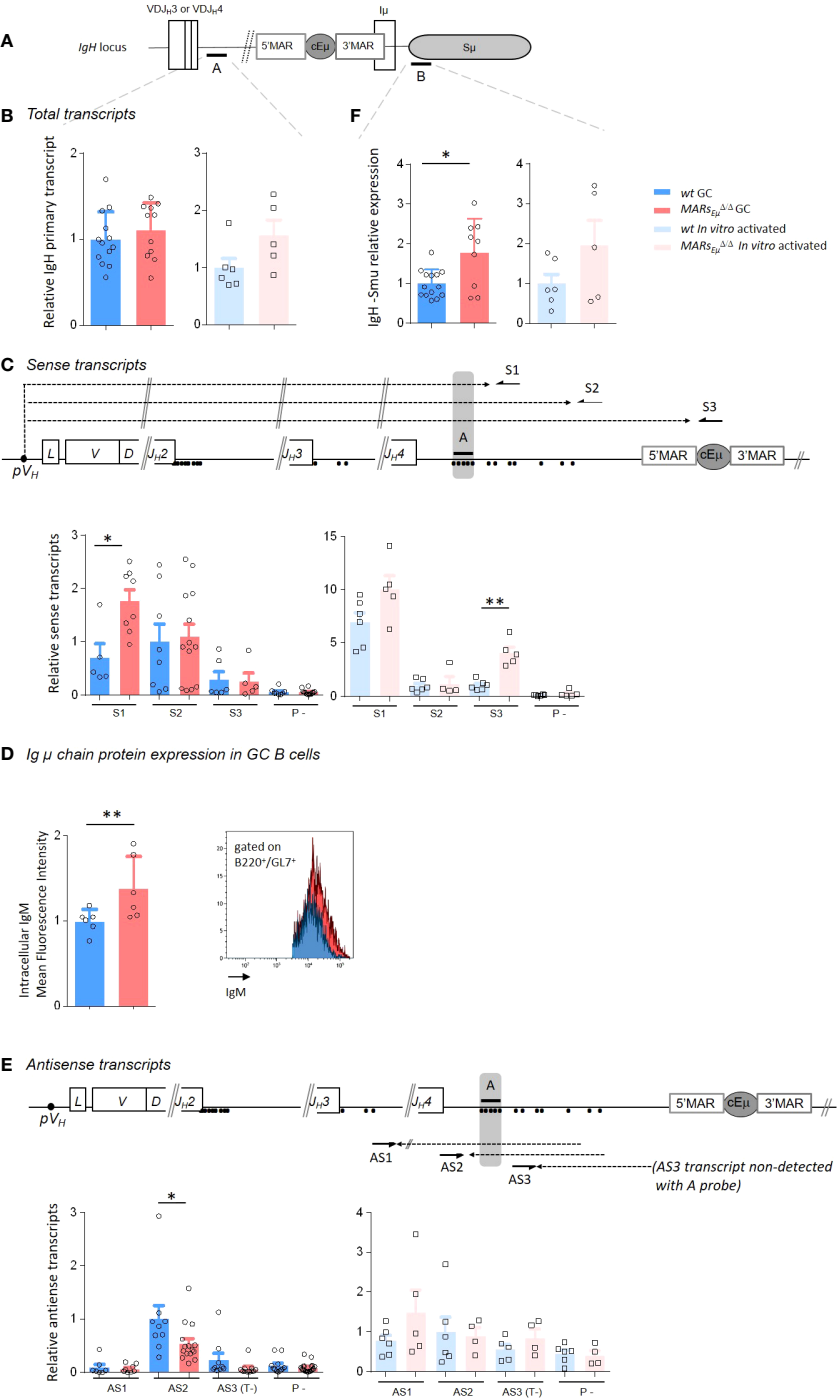
*MARs_Eµ_* deletion impairs strand-specific transcription upstream from *Eµ* region. **(A)** *IgH* locus specifying location of q-PCR probes **(A, B)** used for transcripts quantification. **(B)** Total primary transcripts quantified downstream from the *J_H_4* segment with probe A in Peyer’s patch GC B cells (dark colors) and *in vitro*-activated B cells (light colors) from *wt* and *MARs_Eµ_* ^Δ/Δ^ mice. **(C)** Detection of sense transcripts (dotted arrows) in murine *IgH* locus (not to scale). Arrows indicate primers (S1, S2, S3) downstream from *J_H_3 and J_H_4* used for strand-specific reverse transcription. Primary sense transcripts were quantified with probe A (black bar) in Peyer’s patch GC B cells and *in vitro*-activated B cells from *wt* and *MARs_Eµ_* ^Δ/Δ^ mice. Dots indicate antisense transcript start sites according to Perlot et al. (38). Baseline levels were defined by using a RT template performed without primers (P-). Bar graphs show the relative quantity of sense transcripts obtained from template S1, S2 and S3 (mean ± SEM) from two to three independent experiments. **(D)** Intracellular IgM mean fluorescence intensity measured by flow cytometry in GC B cells from Peyer’s patches of *wt* and *MARs_Eµ_* ^Δ/Δ^ mice. Bar graphs indicate data from individual mice (*n*=6 mice in 2 independent experiments, mean ± SEM); a representative example of cell count overlay is shown. **(E)** Detection of antisense transcripts (dotted arrows) in murine *IgH* locus (not to scale). Arrows indicate primers (AS1, AS2, AS3) downstream from *J_H_3 and J_H_4* used for strand-specific reverse transcription. Primary antisense transcripts were quantified with probe A (black bar) in Peyer’s patch GC B (dark colors) cells and *in vitro*-activated (light colors) B cells from *wt* and *MARs_Eµ_* ^Δ/Δ^ mice. Dots indicate antisense transcripts start sites according to the Alt study (38). Baseline levels were defined by using a RT template performed without primers (P-) or by using a strand-specific template that cannot be detected with A probe (T-). **(F)** Total primary transcripts quantified downstream from *cEµ* region with probe B in Peyer’s patch GC B cells (dark colors) and *in vitro*-activated B cells (light colors) from *wt* and *MARs_Eµ_* ^Δ/Δ^ mice. p-value was determined with two tailed Mann Whitney test; significant differences are indicated by: *P < 0.05; **P < 0.01 and error bars represent SEM of two to three independent experiments.

It is well established that SHM in Ig *V* segments is coupled to transcription initiated at *V* promoters (36). To investigate transcription-related events in SHM-targeted regions upstream and downstream from *Eµ*, we precisely quantified the total amounts of steady state total *IgH* primary transcripts by using multiple q-PCR probes located respectively downstream from *J_H_4* and *J_H_3:* the previously described probe A (37) (**Figure 3A** and **Supplementary Figure S4**) complemented by probes A’ and C (**Supplementary Figures S4**, **S5**). The use of cDNA templates conducted with random hexamers showed that the amount of total IgH primary transcripts running upstream from *Eµ* did not display significant variations between *wt* and *MARs_Eµ_*-deficient cells in both GC and *in vitro*-stimulated samples by using probe A (**Figure 3B**) as well as with probes A’ and C, although an upward trend could be noticed in LPS-activated samples (**Supplementary Figure S5A** left). The intriguing discrepancy between the mutation phenotype observed in *MARs_Eµ_*-deficient GC B cells and the silent effect on global transcription motivated a more complete study of transcription events occurring upstream from *Eµ*, particularly sense and antisense transcription since the latter has been found in cells undergoing SHM (38). To proceed, we generated cDNA templates with sense transcripts, initiated at the promoter of the rearranged *VDJ* segment, with three primers located downstream from the *J_H_4* segment (S1 and S2) and within the *cEµ* enhancer (S3) (**Figure 3C** and **Supplementary Figure S4**, **S5A**). Reciprocally, we generated cDNA templates with antisense transcripts, initiated in the intronic regions upstream from *Eµ* as described by Perlot et al. (38), with four primers respectively located downstream from *J_H_2* (AS0), *J_H_3* (AS1) and *J_H_4* (AS2 and AS3) (**Figure 3E** and **Supplementary Figure S4** and **S5B**). For both sense and antisense, quantification of steady state transcripts was possible with the same probes A, A’ and C. For strand-specific quantification assays with a given probe, the baseline level was either provided by a control reaction (P-) measuring endogenous priming since devoid of primer or by one strand-specific template that cannot be detected by the probe (T-) as reported previously (39, 40). To note, strand-specific transcripts were optimally detected when primers and probes were closer (sense transcripts with primer S1/probe A or antisense transcripts with primer AS2/probe A). Side by side comparison of *wt* and *MARs_Eµ_*-deficient activated B cells samples revealed several interesting differences. When quantified with optimal primer S1/probe A tandem, sense transcripts were significantly increased in the absence of *MARs_Eµ_* (**Figure 3C**). In GC B cells, a two fold increase was noticed by using S1 template (**Figure 3C**, left bar graph, p=0.019). In *in vitro*-activated cells, an increase of sense transcription was also observed upon *MARs_Eµ_*-deletion, this effect became significant for long transcripts that reach the *cEµ* (**Figure 3C**, right bar graphs, p=0.004 for S3/probe A). By using A’ and C probes, sense transcripts were hardly detectable in GC samples (**Supplementary Figure S5A**; middle bar graphs); although a significant increase was noticed with S3/probe C (p=0.043) and S2/probe C (p=0.02) tandems in LPS-activated samples upon *MARs_Eµ_*-deletion (**Supplementary Figure S5A**; right bar graphs). As a potential consequence of the increased transcription of the *VDJ* unit in observed upon *MARs_Eµ_* deletion, we measured by flow cytometry the level of intracellular Ig*µ* chain in Peyer’s patch GC B cells (B220^+^/GL7^+^) of *wt* and *MARs_Eµ_* ^Δ/Δ^ mice. The significant increase of Ig*µ* chain expression in the absence of *MARs_Eµ_* region (**Figure 3D**, p=0.002) corroborate our sense-transcription data. This indicated that the absence of *MARs_Eµ_* certainly did not hamper RNA pol II machinery to progress 3’ to the *VDJ* unit and might even facilitate this process in activated cells.

Globally less abundant than their sense counterparts, quantification of antisense transcripts running downstream from *J_H_* segments showed quite different patterns (**Figure 3E** and **Supplementary Figure S5B**). While quite similar levels were detected in LPS-activated samples (**Figure 3E** right and **Supplementary Figure S5B**), intronic antisense transcripts were about 2 fold less abundant in *MARs_Eµ_*-deficient GC B cells when detection was allowed by optimal probe/primer combination (**Figure 3E**, left, p=0.025, AS2/probe A and **Supplementary Figure S5B** left, p=0.041 for AS3/probe A’). As a complementary experiment, sense and antisense transcription patterns were also determined in naive B cells in order to assess whether the influence of MARs was restricted to the activated stage. Data proved this was the case since, in naive cells sorted from Peyer’s patches, transcription patterns as well as intracellular IgM levels were unchanged in *wt* and *MARs_Eµ_* ^Δ/Δ^ mice (**Supplementary Figures S6A, B**); identical transcription patterns were also observed in splenic resting B cells (**Supplementary Figure S6C**).

The obvious unbalanced sense/antisense transcription ratio could result from either weak transcription efficiency or instability of antisense products. Nevertheless, Perlot et *al.* identified by RACE assays, in normal GC B cells, multiple antisense-transcript initiation start sites downstream from every *J_H_* region and raised the question of specific enhancers. Our current data refines this previous study by identifying *MARs_Eµ_* as potential boosters of antisense transcripts that, given their proximity to the enhancer, could achieve some regulatory function like eRNA or PROMPT/uaRNA (41). Highlighting a correlation between mutation efficacy and strand-specific transcription pattern upstream from *Eµ*, our data support the idea that some level antisense transcription downstream from the *VDJ* exon could prepare to SHM (38). Seemingly transient, specific to cell subsets and occurring upstream from an enhancer, such antisense transcripts could be substrates for RNA exosome and lead to optimized SHM targeting as proposed by Basu and colleagues (42–44).

Since a strong increase of mutations was observed within the *Sµ* region in the absence of *MARs_Eµ_*, we also sought to correlate SHM and transcription on the other side of *cEµ* by quantifying total transcripts (probe B) running in this region (**Figure 3A**). In this case and according to what could be expected, transcription was significantly increased in *MARs_Eµ_*-deficient GC B cells from Peyer’s patches (**Figure 3F** left, p=0.04); a similar trend, although not significant, was observed in LPS activated B cells (**Figure 3B** right p=0.32). Accordingly, we also observed a modest but reproducible increase of CSR Cγ3 and Cγ1 in *MARs_Eµ_*-deficient B cells stimulated *in vitro* respectively by LPS or by LPS + IL4 cocktail (**Supplementary Figure S7**). A similar modest CSR effect associated to *Sµ* internal deletions has been previously reported in hybridomas carrying the same *MARs_Eµ_*-deletion (34). This indicated that the absence of *MARs_Eµ_* leads to a global increase in transcription of the donor S region and consequently favours SHM targeting.

The significant changes in transcription patterns upstream and downstream from *cEµ* observed in our models put forward the hypothesis that *MARs_Eµ_* act as physiological barriers in activated B cells, limiting sense transcription of the *VDJ* unit up to the intronic enhancer. For transcription running through the *Sµ* region, our data is in agreement with a repressive function of *MARs_Eµ_* in activated B cells, in order to limit SHM targeting of this area. However, our data also suggest that *MARs_Eµ_* act as transcriptional repressors of the *VDJ* unit in both naïve and activated cells; a statement in contradiction with our hypothesis that *MARs_Eµ_* facilitates SHM upstream from *cEµ*. To settle such a discrepancy in our *MARs_Eµ_*-deficient B cells, we first questioned AID deamination efficiency and second error-prone repair pathways processing in SHM targeted regions: upstream and downstream from *cEµ*.

### *MARs_Eµ_* deletion impairs error-prone repair pathway upstream from the Eµ enhancer region

**Figure 4:**
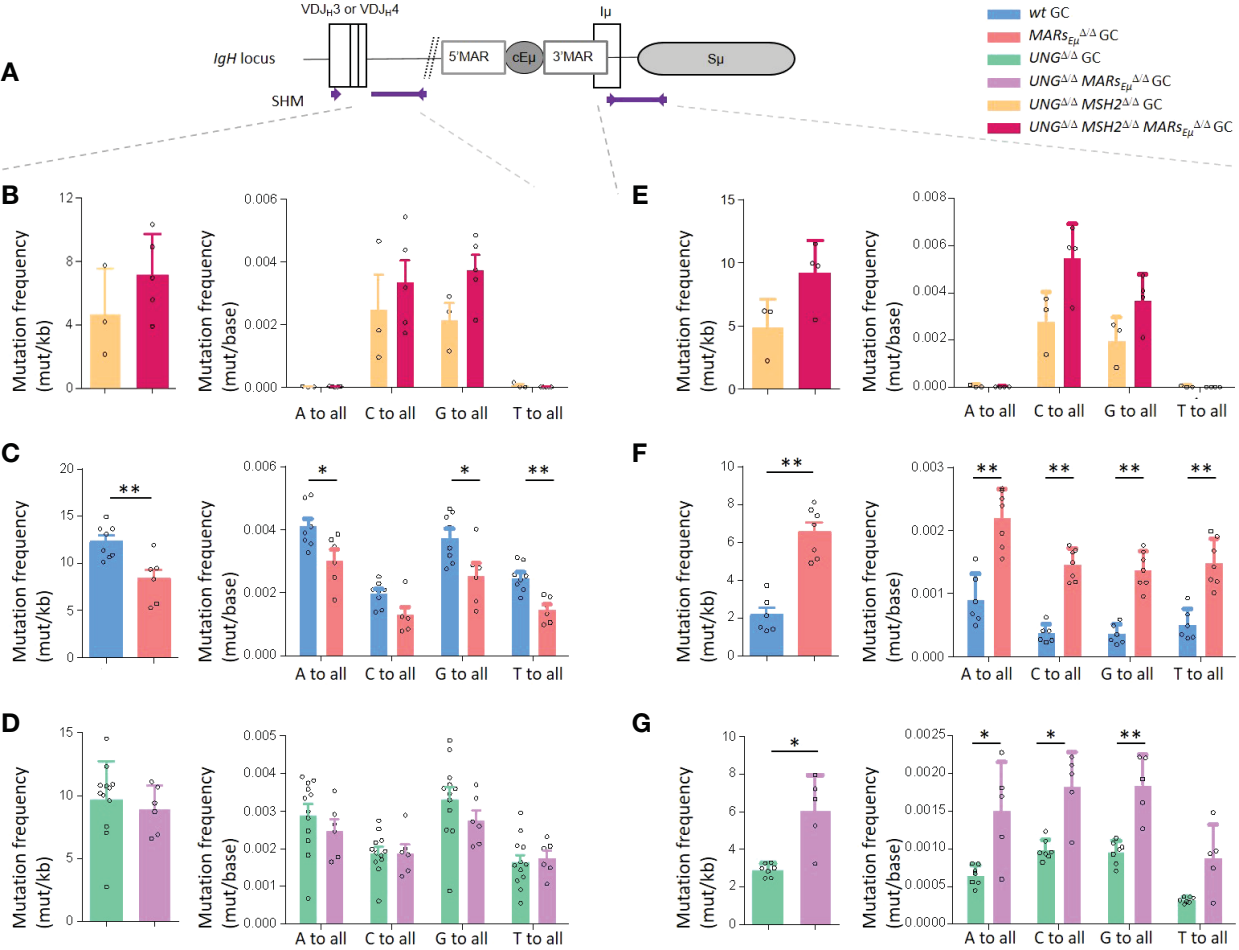
*MARs_Eµ_* deletion impedes error-prone repair pathways upstream from *Eµ* region. Comparison of *IgH* SHM events occurring on both sides of *cEµ* in Peyer’s patch GC B cells sorted from *wt* and *MARs_Eµ_* ^Δ/Δ^ mice models, bred in genetic backgrounds deficient for base excision repair (*Ung* KO) and mismatch repair (*Msh2* KO). Data were obtained by NGS (Ion Proton) combined to DeMinEr filtering (28). In each region, analyzed and represented as a panel, bar graphs report overall mutation frequencies (left) and detailed mutation frequencies at all bases (right). **(A)** Location of *IgH* regions (thick purple lines) tested for SHM, arrows represent primers used for PCR amplification. **(B)** SHM downstream from *J_H_4* in double-deficient *Ung^Δ/Δ^ Msh2^Δ/Δ^* background. **(C)** SHM downstream from *J_H_4* in DNA repair proficient (*Ung^+/+^ Msh2^+/+^*) background. **(D)** SHM downstream from *J_H_4* in *Ung^Δ/Δ^* background. **(E)** SHM downstream from *cEµ* in double-deficient *Ung^Δ/Δ^ Msh2^Δ/Δ^* background. **(F)** SHM downstream from *cEµ* in DNA repair proficient (*Ung^+/+^ Msh2^+/+^*) background. **(G)** SHM downstream from *cEµ* in *Ung^Δ/Δ^* background. p-value was determined with two tailed Mann Whitney test; significant differences are indicated by: *P < 0.05; **P<0.01 and error bars represent SEM of two to three independent experiments.

One critical experiment needed to challenge the function of *MARs_Eµ_* as physiological barrier for SHM machinery was to first assess whether IgH AID targeting could be impaired in the absence of *MARs_Eµ_*. To proceed, we bred our *MARs_Eµ_*-KO mice in a genetic background deficient for both base excision repair (*Ung* ^Δ/Δ^) and mismatch repair (*Msh2* ^Δ/Δ^) in order to evaluate, on and unbiased manner, the DNA footprint of AID deamination upstream and downstream from *cEµ* (**Figure 4A**). As expected and according to the literature (45–47), models deficient for both BER and MMR displayed only transitions at C/G pairs reflecting cytidine deamination on respectively the template and non-template strands. By looking at deamination frequencies between control (*Ung* ^Δ/Δ^ *Msh2* ^Δ/Δ^) and mutant animals (*Ung* ^Δ/Δ^ *Msh2* ^Δ/Δ^ *MARs_Eµ_* ^Δ/Δ^), our data showed that AID activity upstream from *cEµ* was not impeded upon *MARs_Eµ_*-deletion; while differences were not statistically significant (evaluated on n=3 to 4 mice of each genotype), cytidine deamination even tended to be increased in B cells devoid of *MARs_Eµ_*, on both sides of *cEµ* (**Figure 4B** and **Table S4A**). When compared to control animals (*Ung* ^Δ/Δ^ *Msh2* ^Δ/Δ^), nucleotide substitution patterns were unchanged in the absence of *MARs_Eµ_* (**Figure 4B** and **Supplementary Figure S8A**), proving identical strand-specific cytidine deamination: roughly 2/3 on the non-template strand (C to T substitutions) and 1/3 on the template strand (G to A substitutions). Besides imbalanced transcription upstream from *Eµ*, this data indicates that *MARs_Eµ_*-deletion does not impact the choice of any DNA strand for AID targeting within intronic regions.

This notable increased AID deamination footprint prompted by *MARs_Eµ_*-deletion was in total agreement with the increased transcription observed in the corresponding regions of activated B cells. The obvious discrepancy between efficient C to U deamination events and the strong SHM targeting defect within the same *J_H_* intron region unravel the origin of the SHM defect in *MARs_Eµ_*-deficient mice as a default of the mutagenic process occurring downstream from the normally-introduced U-G mismatches in DNA.

This prompted us to investigate whether *MARs_Eµ_*-deletion could provoke skewed mutation patterns within SHM-targeted regions. In the intron region downstream from *J_H_4*, mutation frequency at each of the four bases in *wt* and *MARs_Eµ_*-deficient backgrounds (**Figure 4C** and **Table S2A**) revealed a global significant decrease of mutations at all bases except for substitutions occurring at C in the absence of *MARs_Eµ_*. Similarly, beyond significant differences for C>A, G>C, T>A and T>G events, individual mutation patterns unveiled a global decrease that did not offer any clear hypothesis regarding the mechanism impeding SHM upon *MARs_Eµ_*-deletion. Factually, the unchanged relative proportion of transitions and transversions at C:G base pairs in *MARs_Eµ_* deficient cells did not suggest any defect in UNG activity (45, 48, 49) (**Supplementary Figure S8B**).

To solve this paradox, we bred our *MARs_Eµ_*-KO mice into base excision repair deficient background (*Ung* ^Δ/Δ^) and analysed SHM in the same region. Strikingly, in the absence of UNG, SHM frequency within the *J_H_4* intron region was identical upon the presence (*Ung* ^Δ/Δ^ control mice) or the absence of *MARs_Eµ_* (*Ung* ^Δ/Δ^ *MARs_Eµ_* ^Δ/Δ^ mice) (**Figure 4D** and **Supplementary Table S4B**). Beyond the expected increase of G/C transitions, a typical hallmark of UNG-deficient background, substitution frequencies at all four bases were also identical in *Ung* ^Δ/Δ^ *MARs_Eµ_* ^Δ/Δ^ mice (**Figure 4D**) the same was true when looking at individual substitution events (**Supplementary Figure S8C**). The fact that the SHM deficiency induced by *MARs_Eµ_* deletion was no more observed in UNG-deficient background (**Figures 4C, D**) strongly imply the involvement of BER pathway in the initial mutagenic defect. This same data also proved that SHM events occurring independently of UNG (*e.g*. altogether obtained by replication across U and/or processed by MMR pathway) took place normally within the *J_H_* intron in the absence of *MARs_Eµ_*. Given this, a rational hypothesis to explain the origin of the SHM defect in our model was that abasic sites generated by UNG upstream from *cEµ* are processed differently upon the absence of *MARs_Eµ_*. Our data suggests that U:G mismaches processed by UNG are accurately repaired in the absence of *MARs_Eµ_* while these are normally subject to error-prone repair; sustaining for a specific function of *MARs_Eµ_* in recruiting mutagenic factors associated to BER.

In contrast to what observed in the *J_H_* intron, substitution frequencies and mutation patterns downstream from *cEµ* evidenced a different function for such regulatory regions. In B cells capable of BER and MMR, the absence of *MARs_Eµ_* significantly boosted mutations at all bases by at least two fold (**Figure 4F**), this was true for any kind of substitution (**Supplementary Figure S8E**). Substitution patterns collected in mutant animals devoid of BER and MMR highlighted a global “overtargeting” of the *Sµ* region induced by *MARs_Eµ_* deletion (**Figure 4E**, **Supplementary Figure S8D** and **Supplementary Table S4C**). This was in line with the general increase in both *Sµ* germline transcription observed in this model. In models impaired for BER, our data showed that UNG-deficiency combined to deletion of MARs (*Ung* ^Δ/Δ^ *MARs_Eµ_* ^Δ/Δ^ mice) maintained the SHM burden downstream from *cEµ* significantly higher than what observed for UNG alone (*Ung* ^Δ/Δ^ control mice) (**Figure 4G**; **Supplementary Figure S8F** and **Supplementary Table S4D**). Such a comparison suggests that error-prone repair factors could more readily access to abasic sites generated in the S region when *MARs_Eµ_* are missing. In this way, our data support the idea that *MARs_Eµ_* act as physiological barrier that optimize SHM upstream from the *Eµ* region and rationalize the fact that MARs are evolutionary conserved downstream from Ig gene V regions (24); and moreover conserved structures in mammals (2).

## Concluding remarks

One simplistic model would argue that the most important regulatory regions for *IgH* locus expression are conserved upon any reshaping event occurring in developing B lineage cells (VDJ recombination, CSR and SHM). Beyond the major enhancer regions, *e.g. cEµ* and the *3’RR*, our current study identifies *MARs_Eµ_*, also conserved upon any rearrangement, as part of these most critical *IgH* elements. Since flanking *cEµ*, *MARs_Eµ_* could potentially interfere with the function of this regulatory element. Our current study showed that *MARs_Eµ_* deletion impact the transcription pattern in this region but also points out, when compared to deletion of *cEµ* alone (7), that the core enhancer and *MARs_Eµ_* act independently on SHM. Our data indicate that IgH *MARs_Eµ_* delimit, upstream from the enhancer region, some error-prone repair processes coupled to BER.

Several studies indeed proposed that *J-C* intronic MARs help generate negative supercoiling and consequently increased ssDNA and potential other secondary structures that could promote accessibility to AID (50–52). The hypothesis that *MARs_Eµ_* add again more DNA strain to the sense-transcribed *VDJ* transcription unit is relevant to the positive effect of topoisomerase depletion on AID targeting and SHM (51, 53, 54). In line with transcription dynamics, RNA pol II stalling (55, 56) and DIVAC regulatory regions are being proposed to facilitate AID targeting for SHM (57, 58). A model proposed by Alt and colleagues (59) would be that the optimal chromatin environment for AID-induced mutations would be provided by convergent transcription as the result of fine balance between sense and antisense events. As possible byproducts of RNA Pol II collision, antisense or regulatory transcripts in such regions remain transient and difficult to detect in a *wt* context; probably because processed by RNA exosome or other RNAse activities (60, 61). In line with our data, a recent study from the Basu group emphasize the critical function of the RNA exosome for the fine tuning of sense transcription of the VDJ exon, leading to modifications on strand-specific AID targeting and SHM (44). While also modifying sense and antisense transcription pattern, our study shows that *MARs_Eµ_* deletion does not impede AID footprint but rather some of repair mechanisms acting downstream from the U-G mismatch. Since we hypothesize that UNG activity by itself is not necessarily impaired in our model, our data strongly suggest that only BER-dependent error-prone repair is impeded by *MARs_Eµ_* deletion. This statement supposes that efficient MMR-dependent error-prone repair in this region does not require such a barrier. The question of MARs binding factors and their respective dynamic association to such regulatory regions needs to be further investigated. The literature already suggest that some of them, like the Special AT-rich binding factor 1 (SATB1), could act as accessory factors in BER (62). Recent findings, showing that UNG2-interacting protein FAM72A promotes error prone processing of U-G mismatch in Ig genes (48, 49), raise the question of its specific recruitment to AID-targeted regions; our current study suggests that *MARs_Eµ_* could potentially interact with error-prone factors acting downstream from UNG2. Another future challenge remains to define whether some components of the nuclear matrix, nuclear filaments or proteins anchored in the envelope, could be involved in the anchorage of SHM targets.

## Material and methods

### Mouse models

To generate *MARs_Eµ_* KO model, gene targeting for matrix attachment regions flanking the *IgH Eµ* enhancer element was performed by homologous recombination, in the murine E14 ES cell line, with a vector kindly provided by Dr. Frederick Alt that permitted replacement of the 995 pb region (including *cEµ* and its flanking *MARs*) by a 220 pb *Hinf*I genomic fragment that reintroduced the *cEµ* enhancer, a 256 bp fragment containing plasmid sequence and a “*loxP*-*pGK-Neo^R^*-*loxP*” cassette (18). Once introduced in the mouse germline, the selection cassette was deleted *in vivo* by *cre-loxP* recombination as previously described (9) to obtain the *MARs_Eµ_* ^Δ/Δ^ *IgH* allele devoid of both 5’ and 3’ *MARs_Eµ_* (respectively 344 pb *Xba*I-*Hinf*I and 426bp *Hinf*I-*Xba*I genomic fragments) (**Figure 1A**). Animal procedures were performed on 8 weeks old male and female mice. All models were created in mixed 129Sv;C57BL/6 background. *Wt*, *MARs_Eµ_* ^Δ/Δ^, *Eµ^Δ/Δ^* (9), *Msh2^Δ/Δ^*, *Ung^Δ/Δ^* (a kind gift of Dr S. Storck) and *Aicda^-/-^* (a kind gift of Pr. T. Honjo) homozygous mice were used for our experiments and maintained at 21–23°C with a 12-h light/dark cycle. All experiments comparing *Wt*, *MARs_Eµ_* ^Δ/Δ^, *Eµ* ^Δ/Δ^ in DNA repair proficient background used systematically control mice carrying two IgH alleles of the same haplotype: IgHa (from 129/Ola strain). Procedures were reviewed and approved by the Ministère de l’Education Nationale de l’Enseignement Supérieur et Recherche autorisation APAFIS#16639-2018090612249522v2.

### Southern blots and PCR analysis of cre-mediated *MARs_Eµ_* deletion

Genomic Southern blots were performed as follows: 20 µg genomic DNA were digested by *Sac*I or *Bam*HI and submitted to electrophoresis on a 0.7% agarose gel. DNA was transfered to nylon membranes (MP Biomedicals) by capillarity. Blots were hybridized with [^32^P]-labeled probes generated by random priming. Hybridization with 5’ probe (0.803 kpb *Sac*I-*Sph*I fragment) and 3’ probe (0.8 kpb *Xba*I-*Bam*HI fragment) located outside the homology arms were used to identify ES cell clones in which *MARs_Eµ_* were replaced by the *loxP*-*pGK-Neo^R^*-*loxP* cassette (**Figure 1A**).

### Total serum Ig quantification by ELISA

Sera were collected at 8 weeks of age from non-immunized *wt* and *MARs_Eµ_* ^Δ/Δ^ mice and analyzed for the presence of different Ig classes and subclasses by ELISA as previously described (63).

### SRBC immunisation

Mice were challenged by intraperitoneal injection with 200µL 50% sheep red blood cell suspension and sacrificed 8 days later to collect GC B cells (B220^+^/GL7^+^) in the spleen. Efficient immunization was assessed by counting GC numbers on spleen frozen sections upon immunofluorescence labelling.

### Immunofluorescence labelling of germinal center structures

Spleens cryosections (10µm) were fixed and permeabilized for 20 minutes in cold acetone at -20°C. Sections were stained with the following primary Abs GL7-FITC (1/50), αB220 APC (1/1000), αIgD-AF594 (1/1000) diluted in PBS/FCS 2%/EDTA 2mM, overnight at 4°C. After 2 washes with PBS, DAPI was added. Images were acquired using an epifluorescent microscope (NIKON). GC structures were identified according to their content in B220+GL7+ cells.

### Flow cytometry and cell sorting

Flow cytometry analysis was performed on LSR-Fortessa cell analyzer (BD Biosciences) on single-cell suspensions from fresh organs. Once washed with 2% fetal calf serum-PBS, lymphoid cells from bone marrow, spleen, peritoneal cavity and Peyer’s patches were labeled with various conjugated Abs: αB220-V450, αCD117-PE, αCD43-PE for bone marrow cells. αB220-V450, αCD21-PE, αCD23-FITC, αIgM total-PE, αIgD-FITC and αCD3e-FITC for splenocytes. αB220-V450, αIgM-PE, αCD5-FITC for peritoneal cavity. αB220-V450, αB220-APC, αIgA-FITC, αIgM-PE, αPNA-FITC, αFAS-PE, αKi67-FITC and GL7-FITC for Peyer’s Patches. (Southern Biotechnology Associates; eBioscience; Sigma and BD Biosciences). Flow cytometry cell sorting was performed on an ARIA 3 (BD Biosciences) apparatus on single-cell suspensions from spleens or Peyer’s patches. Once washed with 2% fetal calf serum-PBS, cells were labeled with PNA, GL7, αB220, and αFAS reagents and sorted based on distinct gates defined as germinal center B cells (B220^+^/GL7^+^ or B220^+^/PNA^High^/Fas^+^).

### Cell culture

Splenocytes were collected, after red blood cells lysis, CD43^+^ cells were depleted using anti-CD43 MicroBeads (Miltenyi Biotec). CD43^-^ splenic B cells were cultured for 3 days at a density of 1x10^6^ cells per mL in RPMI 1640 supplemented in 10% serum calf fetal, sodium pyruvate (Lonza), amino acid (NEAA 100x Lonza) and Penicillin-Streptomycin (Gibco) with 1µg/ml LPS (*In vivo*Gen) alone (for transcription assays) or plus 20ng/ml IL4 (PeproTech) (for CSR experiments).

### SHM assays

SHM analysis within the *J_H_4* intron was either performed by cloning followed by classical Sanger method as described (64) or performed directly on PCR products by next generation sequencing using GS Junior (Roche) or Ion Proton system (Applied Biosystem). SHM within Smu intron was performed by using the following primer SmuF: 5’-AAGGGCTTCTAAGCCAGTCC-3’and SmuR: 5’-TAGCCTGGGTCCCTCCTTAC-3’ and sequenced using GS junior sequencer. For GS Junior, sequencing libraries were prepared according to the manufacturer’s instructions, adaptor sequences were added to the previous amplification primer sequences in order to be compatible with the GS-Junior sequencing technology. Amplifications were performed with Phusion^®^ High-Fidelity DNA Polymerase (New England Biolabs) according to the following program: DNA was denatured 40 s at 98°C and then submitted to 38 cycles consisting of 98°C for 10 s, 68°C for 30 s and 72°C for 30 s, and 1 cycle at 72°C for 10 min. PCR products were first purified using NucleoSpin kit (Macherey-Nagel) followed by Ampure bead purification (Beckman Coulter). PCR products were subjected to “PCR emulsion step” (GS Junior+ emPCR Kit (Lib-A), Roche) and sequenced using GS Junior sequencing kit XL+ (Roche) according to the manufacturer’s instructions. Raw sequences were aligned against reference sequences of *IgHJ_4_*-downstream intron or *Smu* and only full length sequences were kept for mutation analysis. For *IgHJ_4_*, clonally related sequences were removed based on the sequence of VDJ junction (*i.e.* CDR3) similarity. No further filtering steps were implemented in our analysis workflow. Mutations were called on each sequence using pairwise alignment algorithm (from biopython package) and only base substitutions were reported. Mutation frequencies were computed as the ratio between the sum of mutated bases in all complete sequences over the total number of aligned bases. For Ion Proton, sequencing libraries were prepared according to the user guide Ion Xpress**™** Plus gDNA Fragment Library Preparation (Cat. no. 4471269, Life Technologies). Briefly, PCR products (100ng) were fragmented by enzymatic digestion (Ion Shear**™** Plus Reagents Kit, Cat. no. 4471248) and ligated to Barcodes and Adapters (Ion Plus Fragment Library Kit, Cat. no. 4471252). After 200 bp size selection step on E-Gel precast agarose electrophoresis system, final amplification was performed. Raw data were processed using DeMinEr tool as described (28). In the case of studies performed in BER or MMR-deficient backgrounds of mixed 129Sv;C57BL/6 backgrounds, all SHM analysis were done by excluding nucleotides that differ between strains.

### RT-PCR and q-PCR

Total RNA was prepared by using TRIzol reagent (Ambion) procedures. RNA samples were first treated with DNase I (Invitrogen) for 15 min at 25°C. RT was performed on 200 ng of total RNA with random hexamers or with specific primer (10µM) (sequence available in **Supplementary Figure S2**.) using superscript III enzyme (Invitrogen). As control, we performed a reverse transcription without primer to determine the threshold (referred ad P^-^ in bar graphs). Each real-time qPCR reaction was performed in duplicate on 10 ng of RNA equivalent, using TaqMan Universal (except for Sµ quantification we used SYBR green Mastermix (TAKARA)) on StepOnePlus system (Applied Biosystems). Primary transcription at *IgH* locus was quantified as previously described (37) and completed with both a set of primers and q-PCR probes close to *J_H_* segments (listed in **Supplementary Figure 2**.) and a set of primers located 5’ to *Sµ*: Smu-Fw (5’-ACCCAGGCTAAGAAGGCAAT-3’), Smu-Rev (5’-CCTTCCTTCTGCGTATCCAT-3’). Relative mRNA levels were normalized to *Gapdh* transcripts with the appropriate TaqMan probe (Mm99999915_g1, Applied Biosystem). Data were analyzed by comparing threshold cycle (*CT*) values according to the 2^-(δδ^ *^CT^* ^)^ method. The *wt* mice templates used as calibrators were S2 for sense transcripts, AS0 or AS2 for antisense transcripts.

### Statistical analysis

If not specified in the figure legend, Mann Whitney two-tailed tests were used for statistical analysis using GraphPad Prism software (*p<0.05, **p<0.01, ***p<0.001, ****p<0.0001).

## Data availability statement

The datasets presented in this study can be found in online repositories. The names of the repository/repositories and accession number(s) can be found below: https://www.ebi.ac.uk/ena, PRJEB52221.

## Ethics statement

The animal study was reviewed and approved by Procedures were reviewed and approved by the Ministère de l’Education Nationale de l’Enseignement Supérieur et Recherche autorisation APAFIS#16639-2018090612249522v2.

## Author contributions

OAM, MT, MM, AG, MB, SB, CC, CB, JC, EP and SN performed experiments. EP and SN conceived and supervised the study. MM developed the experimental model. OAM, BV, PG, RM, EP and SN wrote the manuscript. All authors contributed to the article and approved the submitted version.

## Supplementary material

Supplementary Figure 1 Gating strategy to test developing B cells in mice. **(A)** Bone marrow B cell populations in *wt* and *MARs_Eµ_* ^Δ/Δ^ mice. Top row: B220+ CD117+ pre-pro B cells were stained with V450–anti-B220, PE–anti-CD117 Abs. Bottom row: B220+ CD43^High^ pro-B and B220+CD43^Low^ pre-B cell populations were stained with V450–anti-B220, FITC–anti-IgM, and PE–anti-CD43 Abs, gated on the IgM-negative population. **(B)** Splenic B cell subsets in *wt* and *MARs_Eµ_* ^Δ/Δ^ mice. Top row: CD21^High^ CD23^Low^ marginal zone and CD21^Low^ CD23^High^ follicular B cell populations were stained with V450-anti-B220, PE–anti-CD21 and FITC–anti-CD23 Abs, gated on B220+ population. Bottom row: IgM^+^IgD^+^ mature B cells were stained with V450-anti-B220, FITC–anti-IgD and PE–anti-IgM Abs. **(C)** Peritoneal cavity B cells in *wt* and *MARs_Eµ_* ^Δ/Δ^ mice. IgM+ CD5+ -B1a and IgM+ CD5- -B1b subsets were stained with V450-anti-B220, PE-anti-IgM, FITC-anti-CD5 Abs, gated on the B220+ population.

Supplementary Figure 2 SHM frequency within *J_H_4* intron. **(A)** SHM downstream from *J_H_3* and *J_H_4* segments in Peyer’s patch GC B cells sorted from *wt* and *MARs_Eµ_* ^Δ/Δ^ mice. For each genotype, pie charts represent distribution of mutated sequences (proportional to the area in each slice, data obtained by Sanger sequencing method) in individually recombined *IgH* alleles. Number of individual clones is reported in the center (after removal of clonally related sequences based on VDJ junction). Each pie chart represent SHM obtained from an individual experiment. Under each pie chart, SHM frequency, sequencing strategy and sample type (individual mice or pool) is indicated. Mean SHM frequency and p values are reported. **(B)** Bar graph displayed numbers of germinal center structures obtained in the spleen of *wt* and *MARs_Eµ_* ^Δ/Δ^ mice upon SRBC-immunization (left). Representative images of spleen section from immunized *wt* and *MARs_Eµ_* ^Δ/Δ^ animals (right) **(C)** Equivalent data representation than reported in **A** for SHM downstream from *J_H_4* segments in splenic GC B cells sorted from SRBC-immunized *wt* and *MARs_Eµ_* ^Δ/Δ^ mice. Mean SHM frequency and p values are reported.

Supplementary Figure 3 SHM frequency downstream from *cEµ* region. **(A)** SHM downstream from *cEµ* region in Peyer’s patch GC B cells sorted from *wt* and *MARs_Eµ_* ^Δ/Δ^ mice obtained by GS junior sequencing. For each genotype, pie charts represent distribution of mutated sequences (proportional to the area in each slice, data obtained by Sanger and GS Junior sequencing method) in individually recombined *IgH* alleles. Number of individual clones is reported in the center (after removal of clonally related sequences based on VDJ junction). Each pie chart represent SHM obtained from an individual experiment. Under each pie chart, SHM frequency, sequencing strategy and sample type (individual mice or pool) is indicated. Mean SHM frequency and p values are reported. **(B)** Left: schematic representation of both *wt* and *MARs_Eµ_* ^Δ^ alleles to illustrate the distance between *cEµ* and *Sµ* region. Blue and red bars indicate the 473bp-designed region to quantify SHM at the same distance from *cEµ* in the two models. Right: Bar graph showing SHM frequency in the corresponding regions of *wt* and *MARs_Eµ_* ^Δ/Δ^ mice, the analysis was performed on samples described in **A**.

Supplementary Figure 4 Annotated nucleotide map of the *IgH*-*J_H_1* to *Eµ* germline region from of 129 wt mice. All *J_H_* exons as well as *coreEµ* element are indicated by bold characters. Start sites for antisense transcripts are reported as (*) according to the Alt study (38). Location of primers used for strand-specific reverse transcription (S1, S2, S3, AS0, AS1, AS2, AS3) are indicated by underlines. TaqMan qPCR amplicons (C, A, A’) are highlighted in grey.

Supplementary Figure 5 Sense and antisense transcripts quantified with *IgH J_H_3* and *J_H_ 4* exons with additional TaqMan probes. **(A)** Murine *IgH* locus (not to scale) indicating location of primers (S1, S2, S3; black arrows) within introns downstream from *J_H_3 and J_H_4* used for strand-specific reverse transcription to detect sense transcripts (dotted arrows). Black bars (A’ and C) indicate location of q-PCR probes. Total primary transcripts and primary sense transcripts were quantified with A’ and C probes in Peyer’s patch GC B cells (dark colors) and *in vitro*-activated B cells (light colors) from *wt* and *MARs_Eµ_* ^Δ/Δ^ mice. **(B)** Murine *IgH* locus (not to scale) indicating location of primers (AS0, AS1, AS2, AS3; black arrows) within introns downstream from *J_H_2, J_H_3 and J_H_4* used for strand-specific reverse transcription to detect antisense transcripts (dotted arrows). Black bars (A’ and C) indicate location of q-PCR probes. Primary antisense transcripts quantified with A’ and C probes in Peyer’s patch GC B cells (dark colors) and *in vitro*-activated B cells (light colors) from *wt* and *MARs_Eµ_* ^Δ/Δ^ mice. Dots indicated antisense transcripts start sites according to the Alt study (38). Baseline level was either provided by using a RT template performed without primers (P-) or using one strand-specific template that cannot be detected with the current probe (T-). p-value was determined with two tailed Mann Whitney test; significant differences are indicated by: *P<0.05; **P<0.01 and error bars represent SEM of two to three independent experiments.

Supplementary Figure 6 Sense and antisense transcription and expression in naive B cells. **(A)** IgH Sense (left bar graph) and antisense (right bar graph) transcripts quantified with A probe in naive B cells sorted from Peyer’s patch in *wt* and *MARs_Eµ_* ^Δ/Δ^ mice. **(A)** Intracellular IgM mean fluorescence intensities measured by flow cytometry in naive B cells from Peyer’s patches of *wt* and *MARs_Eµ_* ^Δ/Δ^ mice. **(C)** IgH sense (left bar graph) and antisense (right bar graph) transcripts quantified with A probe in resting splenic B cells sorted from *wt* and *MARs_Eµ_* ^Δ/Δ^ mice. Bar graphs show mean ± SEM of two independent experiments.

Supplementary Figure 7 Comparison of *in-vitro* Ig class switching in *wt* and *MARs_Eµ_* ^Δ/Δ^ mice. Percentage of IgG3 and IgG1 positive cells measured by flow cytometry after respectively LPS or LPS + IL4 stimulation for 3 days of splenic B cells sorted from *wt* and *MARs_Eµ_* ^Δ/Δ^ mice. Bar graphs show mean ± SEM of one representative experiment for each condition, gating strategy is indicated below.

Supplementary Figure 8 Base substitution patterns in BER- and MMR-deficient backgrounds. Comparison of SHM-related base substitution patterns, reported as frequencies, at *IgH* in Peyer’s patch GC B cells sorted from *wt* and *MARs_Eµ_* ^Δ/Δ^ mice models, bred in genetic backgrounds deficient for base excision repair (*Ung^Δ/Δ^*) and mismatch repair (*Msh2^Δ/Δ^*). Data were obtained by NGS (Ion Proton) combined to DeMinEr filtering (28). **(A)** Substitution pattern downstream from *J_H_4* in double-deficient *Ung^Δ/Δ^ Msh2^Δ/Δ^* background. **(B)** Substitution pattern downstream from *J_H_4* in DNA repair proficient (*Ung^+/+^ Msh2^+/+^*) background. Relative proportions of transitions and transversions at dC/dG pairs are reported in an additional bar graph. **(C)** Substitution pattern downstream from *J_H_4* in *Ung^Δ/Δ^* background. **(D)** Substitution pattern downstream from *Iµ* in double-deficient *Ung^Δ/Δ^ Msh2^Δ/Δ^* background. **(E)** Substitution pattern downstream from *Iµ* in DNA repair proficient (*Ung^+/+^ Msh2^+/+^*) background. **(F)** Substitution pattern downstream from *Iµ* in *Ung^Δ/Δ^* background. p-value was determined with two tailed Mann Whitney test; significant differences are indicated by: *P<0.05; **P<0.01 and error bars represent SEM of two to three independent experiments.

Supplementary Table 1
*MARs_Eµ_* deletion led to normal B-lineage cell development. Bone Marrow and peripheral B cell subsets counts in *wt* and *MARs_Eµ_* ^Δ/Δ^ mice. Absolute numbers are reported as mean± SEM. Significance was assessed with Student T test. P value is indicated when difference is significant.

Supplementary Table 2 SHM data (NGS) from individual mice in DNA repair proficient background. Total number of mutations, total number of bp analyzed and mutation frequencies for *w*t and *MARs_Eµ_* ^Δ/Δ^ mice. **(A)** Data from intron 3’ to *J_H_4* in Peyer’s patches GC B cells, **(B)** Data from spleen GC B cells from SRBC-immunized mice, **(C)** Data from intron 3’ to Iµ in Peyer’s patches GC B cells.

Supplementary Table 3 Unselected SHM within out of frame VDJ exons in GC B cells sorted from Peyer’s patch. Unique VDJ junctions, rearranged to *J_H_3* and *J_H_4* segments, were obtained after amplification, cloning and sequencing by classical Sanger method of DNA extracted from from Peyer’s patch sorted GC B cells (n=2 to 3 independent samples corresponding to individual mice or pools of 7 to 12 mice of each genotype). SHM data were calculated after processing individually recombined IgH alleles by IMGT V-Quest software (https://www.imgt.org/IMGT_vquest); numbers of base substitutions identified as SHM by the software in the FR3 and CDR3 regions are reported in the table and were used to calculate SHM frequencies.

Supplementary Table 4 SHM data (NGS) from individual mice in genetic backgrounds deficient for base excision repair (*Ung*-deficient) and mismatch repair (*Msh2*-deficient). Total number of mutations, total number of bp analyzed and mutation frequencies. **(A)** Data from intron 3’ to *J_H_4* in Peyer’s patches GC B cells of *Ung^Δ/Δ^ Msh2^Δ/Δ^* and *Ung^Δ/Δ^ Msh2^Δ/Δ^* $MARs_{E\mu \;}^{\text{Δ/Δ}}$ mice, **(B)** Data from *Ung^Δ/Δ^* and *Ung^Δ/Δ^* $MARs_{E\mu \;}^{\text{Δ/Δ}}$ mice, **(C)** Data from intron 3’ to Iµ in Peyer’s patches GC B cells of *Ung^Δ/Δ^ Msh2^Δ/Δ^* and *Ung^Δ/Δ^ Msh2^Δ/Δ^* $MARs_{E\mu }^{\Delta }$ mice, **(D)** Data from *Ung* ^Δ/Δ^ and *Ung^Δ/Δ^ MARs_Eµ_* ^Δ/Δ^ mice.
