# Supplementary material for: The IgH Eµ-MAR regions promote UNG-dependent error-prone repair to optimize somatic hypermutation
Source: Front Immunol. 2023 Feb 14;14:1030813. doi: 10.3389/fimmu.2023.1030813 (PMC9971809; doi:10.3389/fimmu.2023.1030813)
Supplement: Supplementary Figure 1 — Gating strategy to test developing B cells in mice. (A) Bone marrow B cell populations in wt and MARsEµ Δ/Δ mice. Top row: B220+ CD117+ pre-pro B cells were stained with V450–anti-B220, PE–anti-CD117 Abs. Bottom row: B220+ CD43High pro-B and B220+CD43Low pre-B cell populations were stained with V450–anti-B220, FITC–anti-IgM, and PE–anti-CD43 Abs, gated on the IgM-negative population. (B) Splenic B cell subsets in wt and MARsEµ Δ/Δ mice. Top row: CD21High CD23Low marginal zone and CD21Low CD23High follicular B cell populations were stained with V450-anti-B220, PE–anti-CD21 and FITC–anti-CD23 Abs, gated on B220+ population. Bottom row: IgM+IgD+ mature B cells were stained with V450-anti-B220, FITC–anti-IgD and PE–anti-IgM Abs. (C) Peritoneal cavity B cells in wt and MARsEµ Δ/Δ mice. IgM+ CD5+ -B1a and IgM+ CD5- -B1b subsets were stained with V450-anti-B220, PE-anti-IgM, FITC-anti-CD5 Abs, gated on the B220+ population. [file DataSheet_1.pdf]

A Bone Marrow

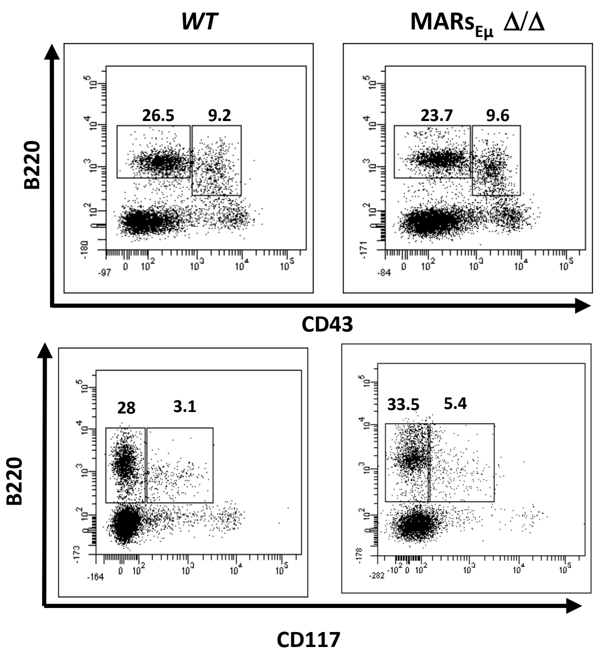

B Spleen

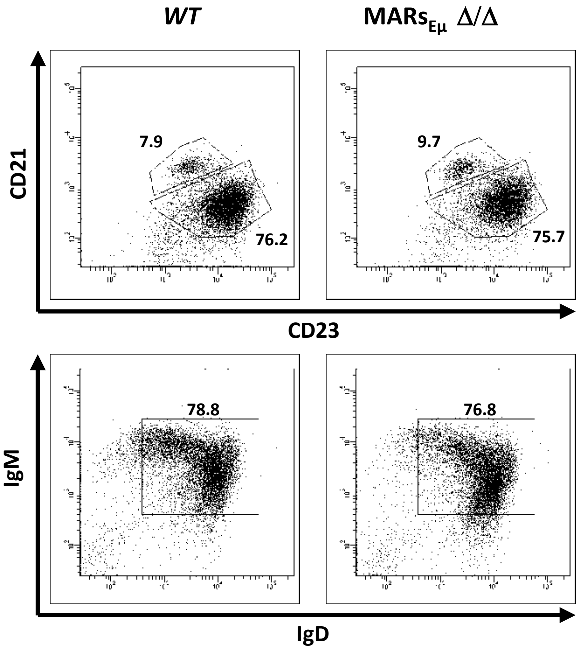

C Peritoneal Cavity

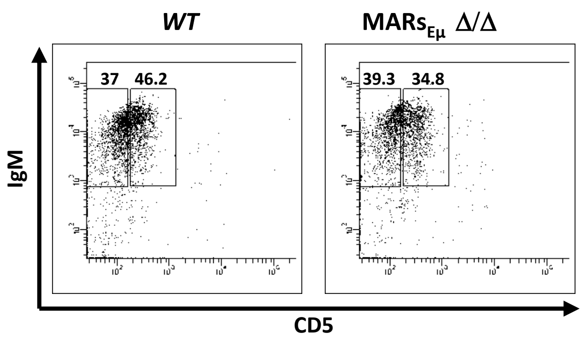

Supp Fig S1

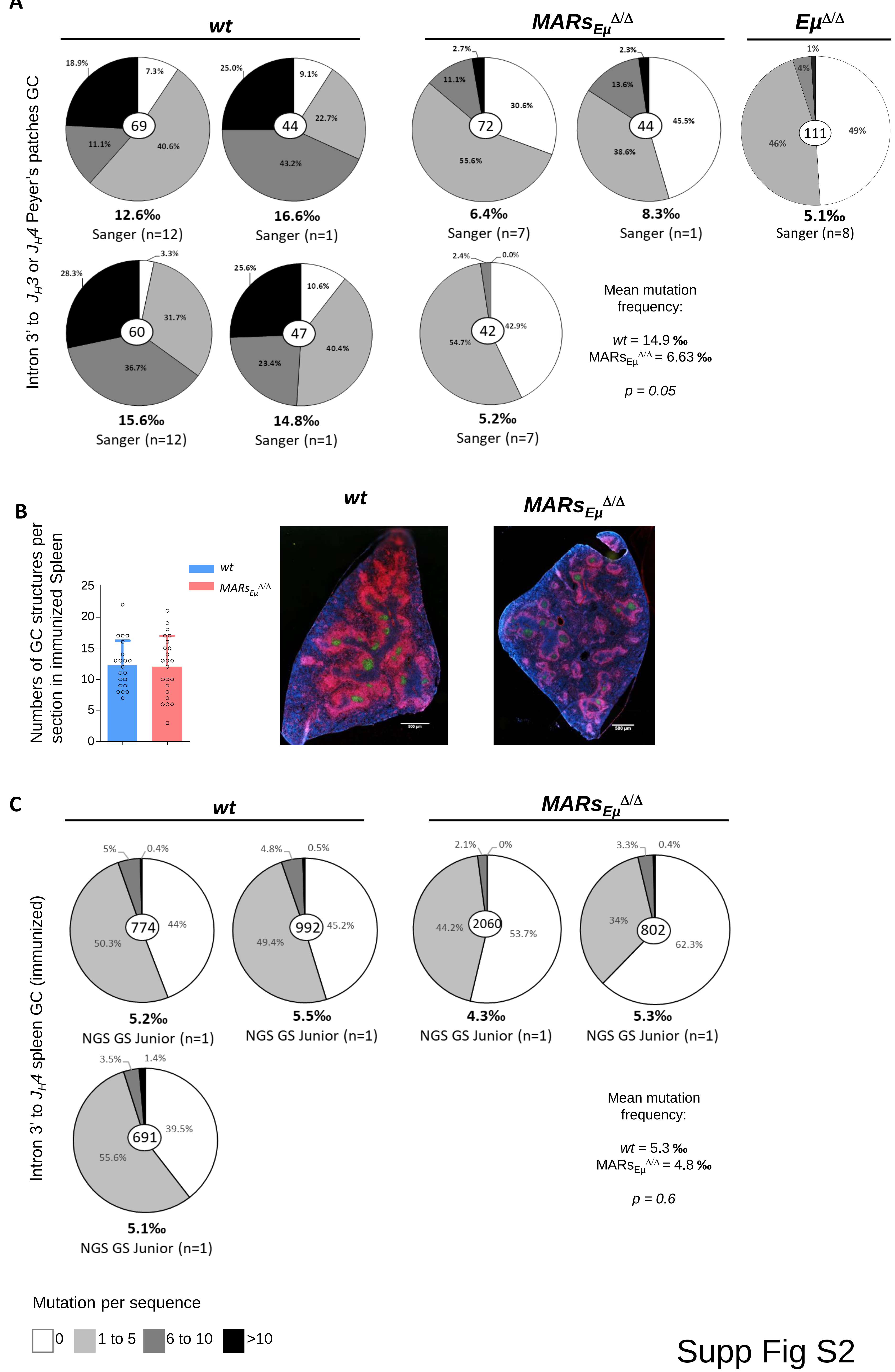
A
wt
MARs$_{Eμ}$$^{Δ/Δ}$
Eμ$^{Δ/Δ}$
Intron 3' to J_H3 or J_H4 Peyer's patches GC
69
18.9%
7.3%
40.6%
11.1%
12.6‰
Sanger (n=12)
44
25.0%
9.1%
22.7%
43.2%
16.6‰
Sanger (n=1)
72
2.7%
11.1%
30.6%
55.6%
6.4‰
Sanger (n=7)
44
2.3%
13.6%
45.5%
38.6%
8.3‰
Sanger (n=1)
111
1%
4%
49%
46%
5.1‰
Sanger (n=8)
60
3.3%
28.3%
31.7%
36.7%
15.6‰
Sanger (n=12)
47
25.6%
10.6%
40.4%
23.4%
14.8‰
Sanger (n=1)
42
2.4%
0.0%
42.9%
54.7%
5.2‰
Sanger (n=7)
Mean mutation frequency:
wt = 14.9 ‰
MARs$_{Eμ}$$^{Δ/Δ}$ = 6.63 ‰
p = 0.05
B
wt
MARs$_{Eμ}$$^{Δ/Δ}$
Numbers of GC structures per section in immunized Spleen
0
5
10
15
20
25
500 μm
C
wt
MARs$_{Eμ}$$^{Δ/Δ}$
Intron 3' to J_H4 spleen GC (immunized)
774
5%
0.4%
44%
50.3%
5.2‰
NGS GS Junior (n=1)
992
4.8%
0.5%
45.2%
49.4%
5.5‰
NGS GS Junior (n=1)
2060
2.1%
0%
44.2%
53.7%
4.3‰
NGS GS Junior (n=1)
802
3.3%
0.4%
34%
62.3%
5.3‰
NGS GS Junior (n=1)
691
3.5%
1.4%
39.5%
55.6%
5.1‰
NGS GS Junior (n=1)
Mean mutation frequency:
wt = 5.3 ‰
MARs$_{Eμ}$$^{Δ/Δ}$ = 4.8 ‰
p = 0.6
Mutation per sequence
0
1 to 5
6 to 10
>10

Supp Fig S2

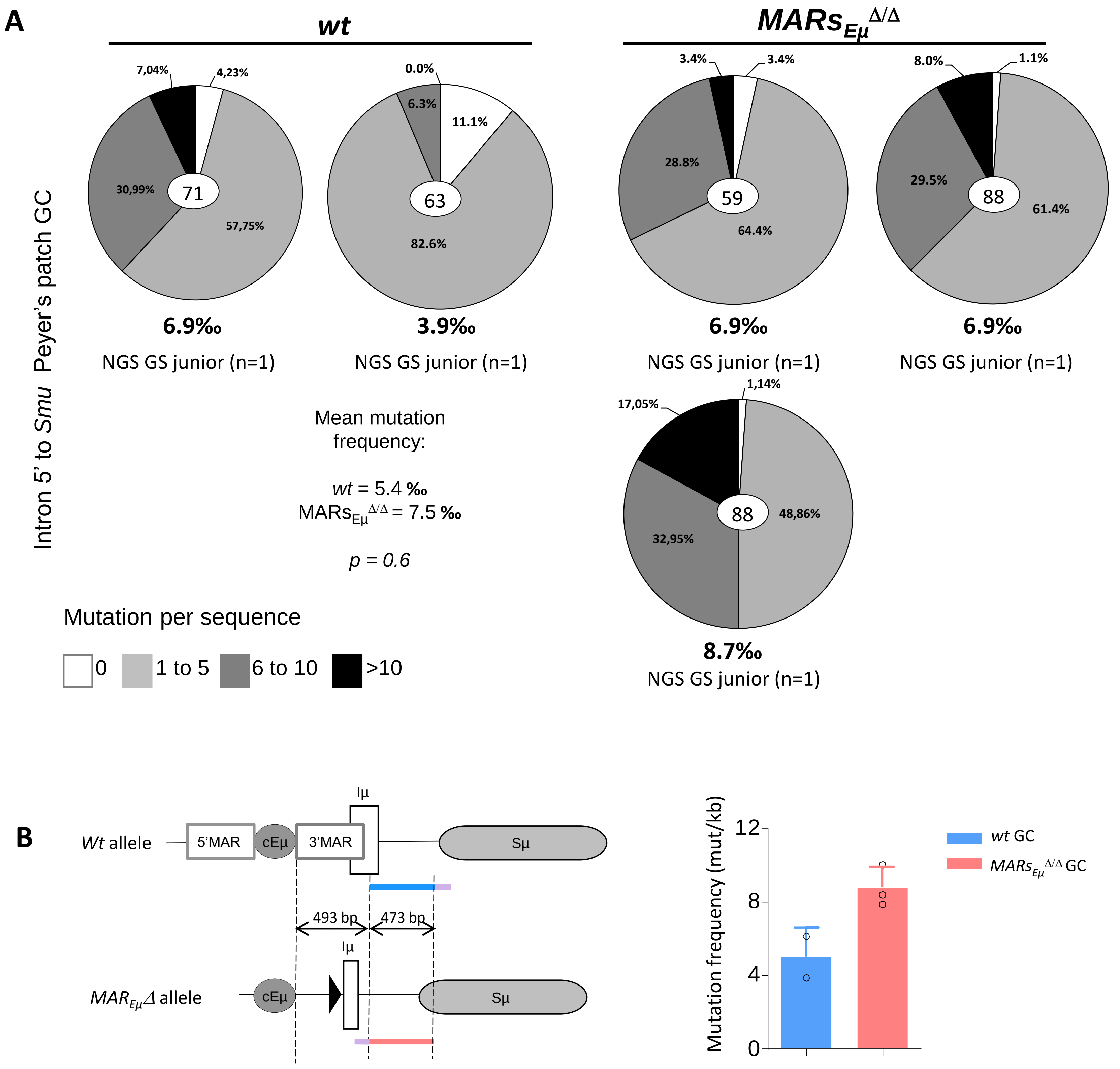
A
wt
MARsEμ Δ/Δ
Intron 5' to Smu Peyer's patch GC
7,04%
4,23%
30,99%
71
57,75%
6.9‰
NGS GS junior (n=1)
0.0%
6.3%
11.1%
63
82.6%
3.9‰
NGS GS junior (n=1)
3.4%
3.4%
28.8%
59
64.4%
6.9‰
NGS GS junior (n=1)
8.0%
1.1%
29.5%
88
61.4%
6.9‰
NGS GS junior (n=1)
Mean mutation frequency:
wt = 5.4 ‰
MARsEμ Δ/Δ = 7.5 ‰
p = 0.6
17,05%
1,14%
88
48,86%
32,95%
8.7‰
NGS GS junior (n=1)
Mutation per sequence
0
1 to 5
6 to 10
>10
B
Wt allele
5'MAR
cEμ
3'MAR
Iμ
Sμ
493 bp
473 bp
MAREμΔ allele
cEμ
Iμ
Sμ
Mutation frequency (mut/kb)
0
4
8
12
wt GC
MARsEμ Δ/Δ GC

Supp Fig S3

J$_H$1

ctactggtacttcgatgtctggggcgcagggaccacggtcaccgtctcctcaggtaagctggctttttctttctgcac
*
attccattctgaaatggaaaagatattctcagatctccccatgtcaggccatctgccacactctgcatgctgcagaag
*
cttttctgtaaggatagggtcttcactcccaggaaaagaggcagtcagaggctagctgcctgtggaacagtgacaatca
*
tggaaaataggcatttacattgttaggctacatgggtagatgggtttttgtacacccactaaaggggtctatgatagtg
tg

J$_H$2 * * * *

actactttgactactggggccaaggcaccactctcacagtctcctcaggtgagtccttacaacctctctcttctattca
* * * * *
gcttaaatagatttactgcatttgttgggggggaaatgtgtgtatctgaatttcaggtcatgaaggactagggacacc
ttgggagtcagaaagggtcattgggagccctggctgatgcagacagacatcctcagctcccagacttcatggccagaga
AS0
tttatagggatcctggccagcattgccgctaggtccctctcttctatgctttctttgtccctcactggcctccatctga
gataatcctggagccctagccaaggatcatttattgtcaggggtctaatcattgttgtcacaatgtg

J$_H$3

cctggtttgcttactggggccaagggactctggtcactgtctctgcaggtgagtcctaacttctcccattctaaatgca
* C
tgttgggggggattctgagccttcaggaccaagattctctgcaaacgggaatcaagattcaacccctttgtcccaaagtt
* * *
gagacatgggtctgggtcagggactctctgcctgctggtctgtggtgacattagaactgaagtatgatgaaggatctgc
cagaactgaagcttgaagtctgaggcagaatcttgtccagggtctatcggactcttgtgagaattaggggctgacagtt
gatggtgacaatttcagggtcagtgactgtctggtttctctgaggtgaggctggaatataggtcaccttgaagacttaa
AS1
gaggggtccaggggggcttctgcacaggcagggaacagaatgtggaacaatgacttgaatggttgattcttgtgtgacac
caggaattggcataatgtctgagttgcccaggggtgattctagtcagactctggggtttttgtcgggtatagaggaaaa
atccactattgtg

J$_H$4

attactatgctatggactactggggtcaaggaacctcagtcaccgtctcctcaggtaagaatggcctctccaggtcttt
* * * AS2
atttttaacctttgttatggagttttctgagcattgcagactaatcttggatatttgtccctgagggagccggctgaga
* * A AS3
gaagttgggaaataaactgtctagggatctcagagccttttaggacagattatctccacatctttgaaaaactaagaatc
* S1 * * *
tgtgtgatggtgttggtggagtccctggatgatgggatagggactttggaggctcatttgagggagatgctaaaacaat
A′
cctatggctggagggatagttggggctgtagttggagattttcagtttttagaataaaagtattagctgcggaatatac
*
ttcaggaccacctctgtgacagcatttatacagtatccgatgcatagggacaaagagtggagtggggcactttctttag
*
atttgtgaggaatgttccacactagattgtttaaaacttcatttgttggaaggagagctgtcttagtgattgagtcaag
*
ggagaaaggcatctagcctcggtctcaaaagggtagttgctg
S2

5′MAR

tctagagaggtctggtggagcctgcaaaagtccagctttcaaaggaacacagaagtatgtgtatggaatattagaagat
gttgcttttactcttaagttggttcctaggaaaaatagttaaatactgtgactttaaaatgtgagagggttttcaagta
ctcatttttttaaatgtccaaaattttgtcaatcaatttgaggtcttgtttgtgtagaactgacattacttaaagttt
aaccgaggaatgggagtgaggctctctcatacccctattcagaactgacttttaacaataataaattaagtttaaaatat
ttttaaatgaattgagcaatgttgagttggagtc

Core Eμ

aagatggccgatcagaaccagaacacctgcagcagctggcaggaagcaggtcatgtggcaaggctatttggggaaggga
S3
aaataaaaccactaggtaaacttgtagctgtggtttgaagaagtggttttgaaacactctgtccagccccaccaaaccg
aaagtccaggctgagcaaaacaccacctgggtaatttgcatttctaaaataagttgag

3′MAR

gattcagccgaaactggagaggtcctcttttaacttattgagttcaacctttaatttagcttgagtagttctagttt
ccccaaacttaagtttatcgacttcaaaatgtatttagaattcattttcaaaattaggttatgtaagaaattgaaggac
tttagtgtctttaatttctaatatatttagaaaacttcttaaaattactctattattcttccctctgattattggtctc
cattcaattcttttccaatacccgaagcatttacagtgactttgttcatgatctttttttagttgtttgttttgccttac
tattaagactttgacattctggtcaaaacggcttcacaaatctttttcaagaccactttctgagtattcattttaggag
aaatactttttttttaaatgaatgcaattatctaga

Supp Fig S4

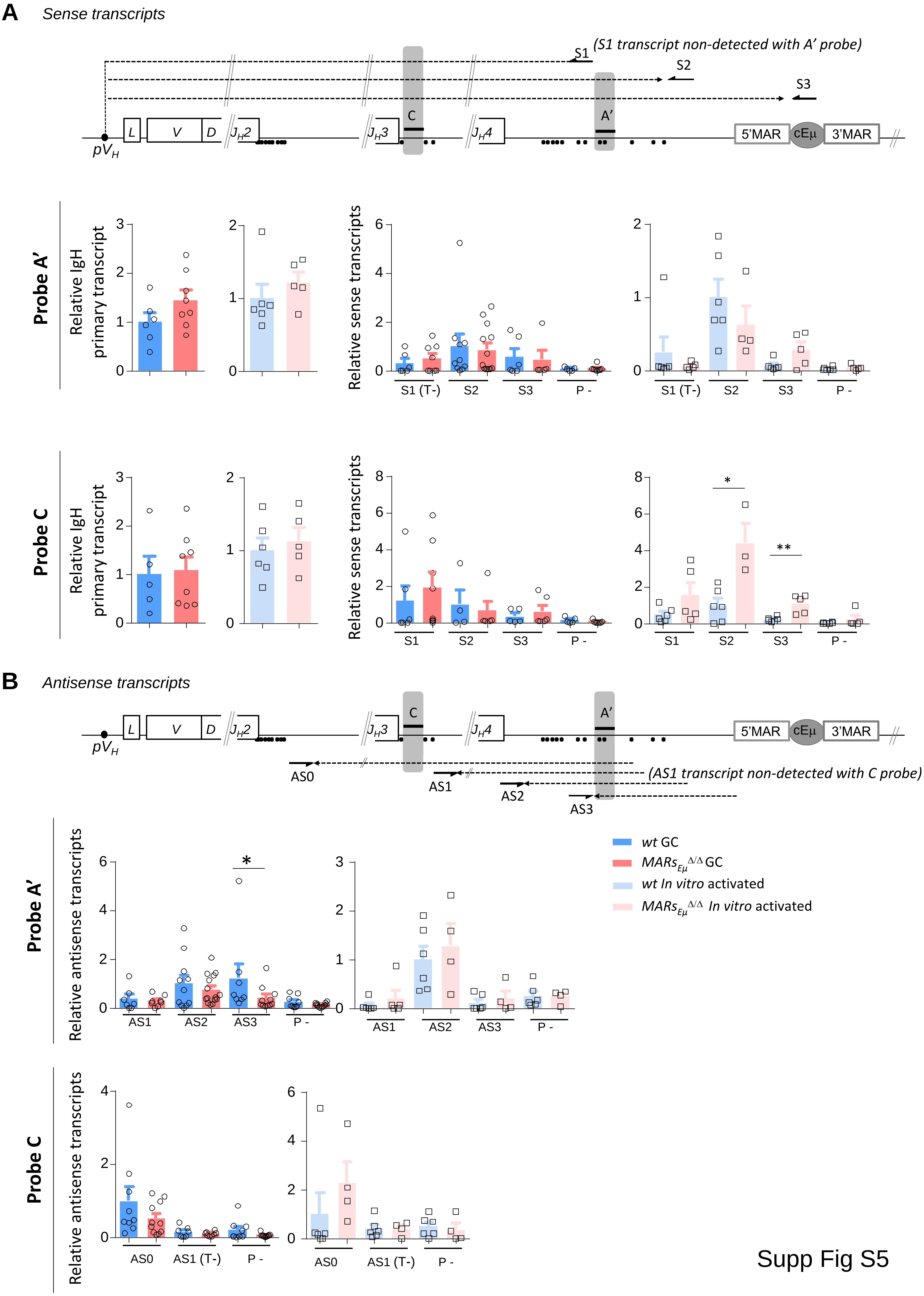
A
Sense transcripts
(S1 transcript non-detected with A' probe)
S1
S2
S3
C
A'
L
V
D
JH2
JH3
JH4
pVH
5'MAR
cEμ
3'MAR
Probe A'
Relative IgH primary transcript
Relative sense transcripts
S1 (T-)
S2
S3
P -
Probe C
S1
*
**
B
Antisense transcripts
AS0
AS1
AS2
AS3
(AS1 transcript non-detected with C probe)
Relative antisense transcripts
AS1 (T-)
wt GC
MARsEμΔ/Δ GC
wt In vitro activated
MARsEμΔ/Δ In vitro activated

Supp Fig S5

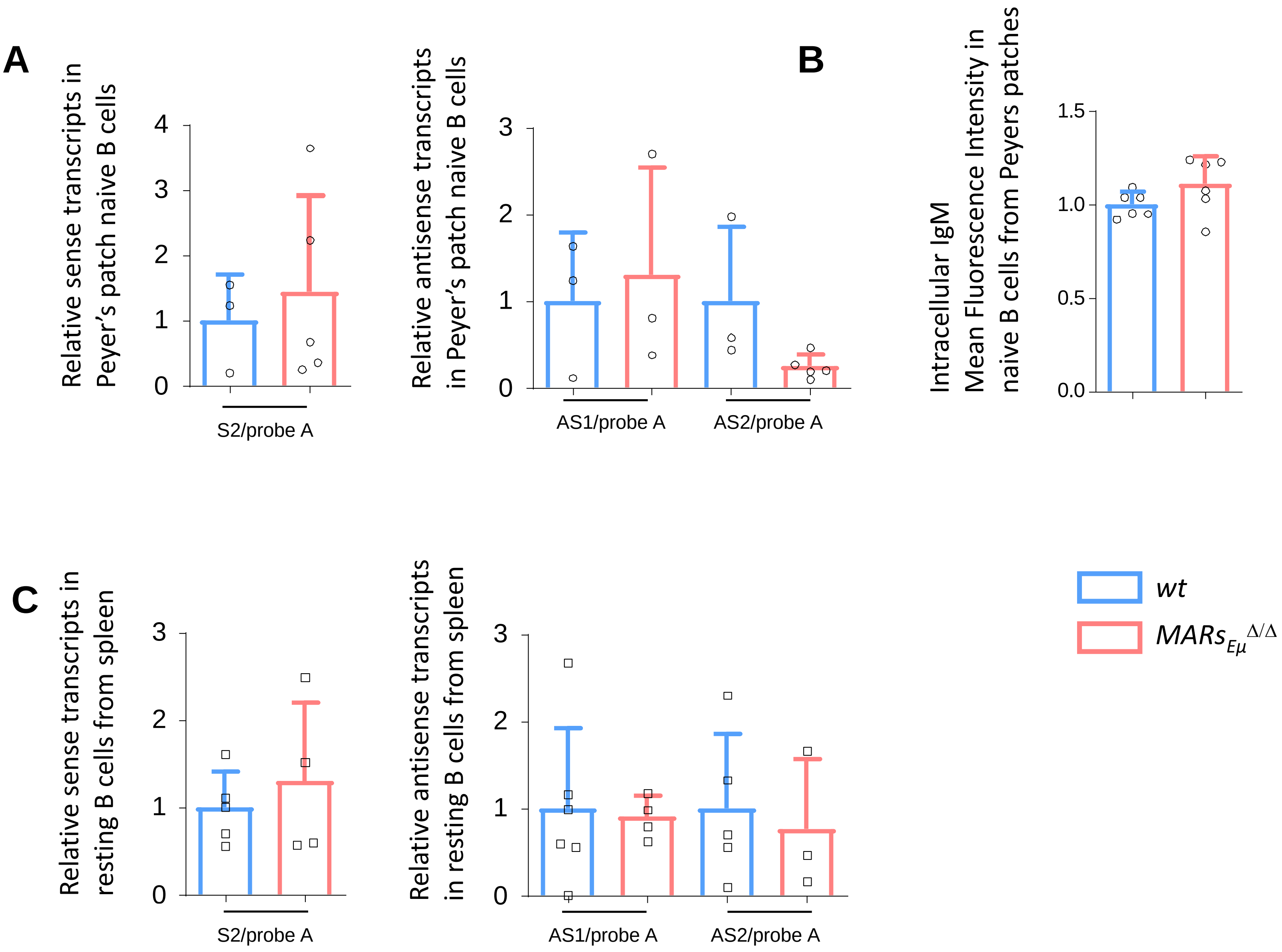
A
Relative sense transcripts in Peyer's patch naive B cells
S2/probe A
Relative antisense transcripts in Peyer's patch naive B cells
AS1/probe A
AS2/probe A
B
Intracellular IgM Mean Fluorescence Intensity in naive B cells from Peyers patches
C
Relative sense transcripts in resting B cells from spleen
S2/probe A
Relative antisense transcripts in resting B cells from spleen
AS1/probe A
AS2/probe A
wt
MARs Eμ Δ/Δ

Supp Fig S6

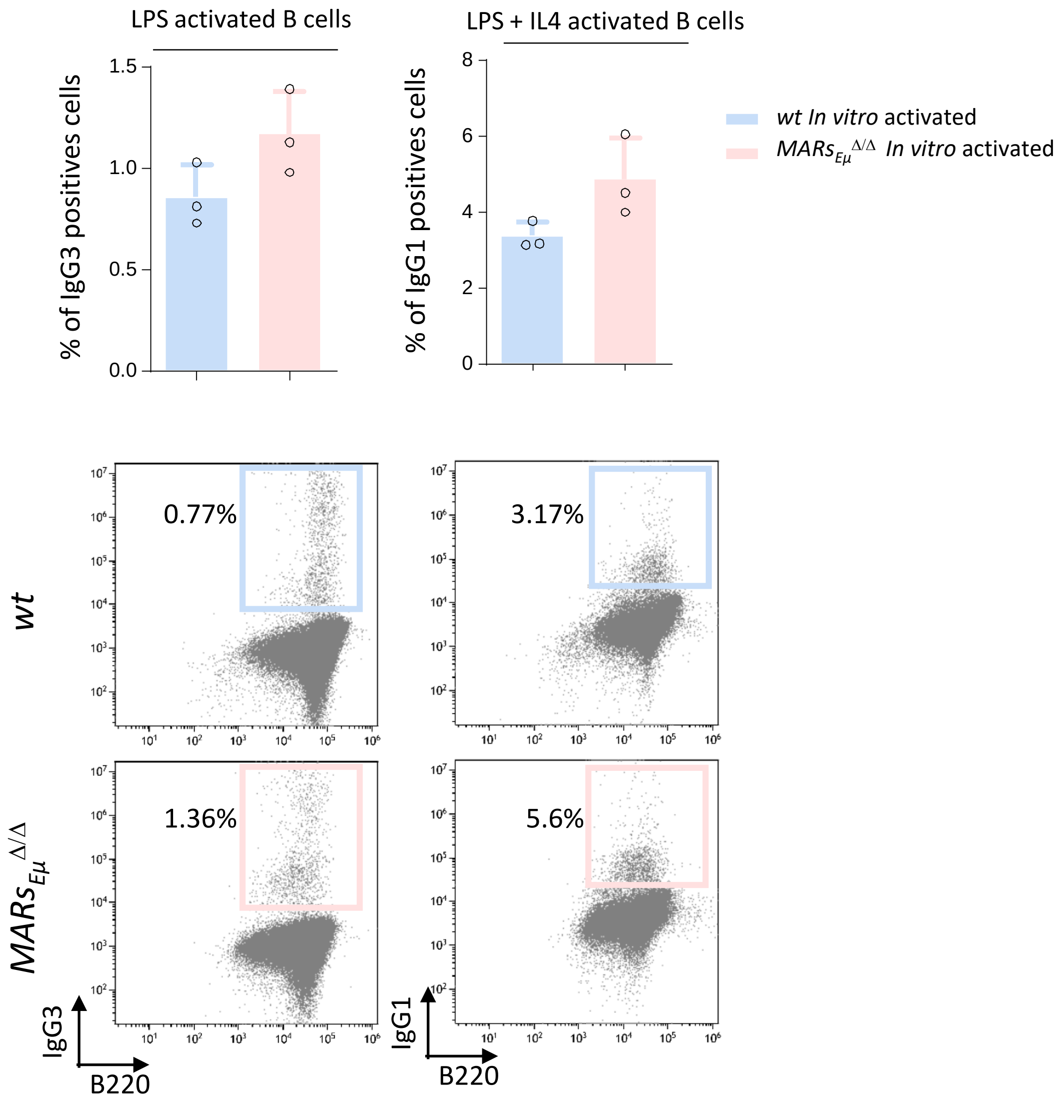

LPS activated B cells
LPS + IL4 activated B cells
% of IgG3 positives cells
% of IgG1 positives cells
wt In vitro activated
MARs Eμ Δ/Δ In vitro activated
wt
MARs Eμ Δ/Δ
0.77%
3.17%
1.36%
5.6%
IgG3
IgG1
B220
B220

Supp Fig S7

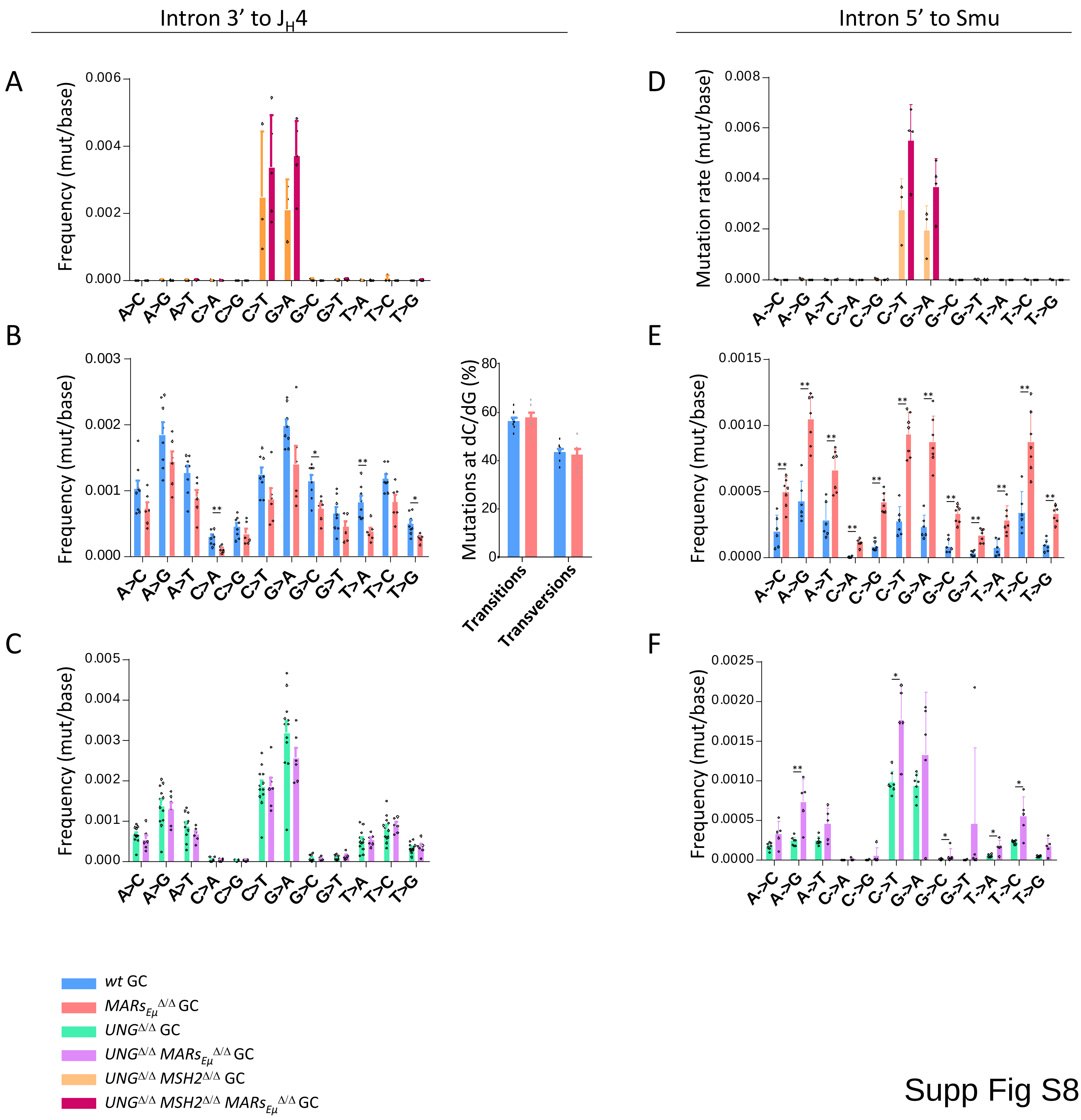

Intron 3’ to JH4
Intron 5’ to Smu
A
B
C
D
E
F
Frequency (mut/base)
Mutation rate (mut/base)
Mutations at dC/dG (%)
Transitions
Transversions
A>C
A>G
A>T
C>A
C>G
C>T
G>A
G>C
G>T
T>A
T>C
T>G
wt GC
MARsEμ Δ/Δ GC
UNGΔ/Δ GC
UNGΔ/Δ MARsEμ Δ/Δ GC
UNGΔ/Δ MSH2Δ/Δ GC
UNGΔ/Δ MSH2Δ/Δ MARsEμ Δ/Δ GC

Supp Fig S8
