# Supplementary material for: The IgH Eµ-MAR regions promote UNG-dependent error-prone repair to optimize somatic hypermutation
Source: Front Immunol. 2023 Feb 14;14:1030813. doi: 10.3389/fimmu.2023.1030813 (PMC9971809; doi:10.3389/fimmu.2023.1030813)
Supplement: Supplementary Table 1 — MARsEµ deletion led to normal B-lineage cell development. Bone Marrow and peripheral B cell subsets counts in wt and MARsEµ Δ/Δ mice. Absolute numbers are reported as mean± SEM. Significance was assessed with Student T test. P value is indicated when difference is significant. [file DataSheet_2.pdf]

Supplementary Table S1

	wt Cell numbers (x10^6)	$MARs_{E\mu}^{\Delta/\Delta}$ Cell numbers (x10^6)	Significance
**Bone marrow**			
B-lineage cells ($B220^+$)	84.96 ± 3.326 N=5	75.27 ± 4.241 N=6	NS
Pre-pro B cells ($B220^+/CD117^+$)	2.183 ± 0.1974 N=6	2.567 ± 0.2951 N=6	NS
Pro-B cells ($IgM^-/B220^+/CD43^{high}$)	7.380 ± 1.131 N=6	7.050 ± 0.9949 N=6	NS
Pre-B cells ($IgM^-/B220^+/CD43^{low}$)	12.50 ± 1.965 N=6	13.54 ± 2.203 N=6	NS
**Spleen**			
B-lineage cells ($B220^+$)	58.16 ± 4.593 N=6	70.68 ± 3.524 N=6	NS
Naïve mature B cells ($B220^+/IgM^+/IgD^+$)	45.09 ± 3.145 N=6	52.76 ± 1.874 N=5	NS
Marginal zone B cells ($B220^+/CD21^{high}/CD23^{low}$)	7.235 ± 1.504 N=6	7.740 ± 0.5829 N=6	NS
Follicular B cells ($B220^+/CD21^{low}/CD23^{high}$)	42.01 ± 3.506 N=6	45.75 ± 2.992 N=6	NS
**Peritoneal cavity**			
B-lineage cells ($B220^+$)	1.864 ± 0.3514 N=5	2.517 ± 0.2998 N=5	NS
B1a cells ($CD5^+/IgM^+$)	0.5924 ± 0.1002 N=5	0.8280 ± 0.1308 N=5	NS
B1b cells ($CD5^-/IgM^+$)	1.076 ± 0.2745 N=5	1.474 ± 0.2010 N=5	NS
**Peyer's patches**			
B-lineage cells ($B220^+$)	4.765 ± 1.106 N=6	5.841 ± 1.172 N=6	NS
$B220^+$ /IgA^+ cells	0.5625 ± 0.1273 N=6	0.8837 ± 0.2218 N=6	NS
$B220^+/IgM^+$ cells	2.828 ± 0.5269 N=6	3.782 ± 0.8263 N=6	NS
Naive B cells ($B220^+/PNA^{low}/Fas^{low}$)	3.684 ± 0.6369 N=5	3.556 ± 0.5026 N=5	NS
Germinal centre B cells ($B220^+/PNA^{High}/Fas^{High}$)	1.044 ± 0.8822 N=8	3.258 ± 1.2 N=11	P=0.0007

Supplementary Table S2

A

	Intron 3' to JH4 Peyer's patch GC					
	wt mice			MARsEμ$^{\Delta/\Delta}$ mice		
# of individual mice	number of mutations	total number of bp analyzed	Frequency (mutation/Kb)	number of mutation	total number of bp analyzed	Frequency (mutation/Kb)
#1	5 919 179	434 026 896	**13.6**	1 835 118	193 099 971	**9.5**
#2	1 381 154	127 267 013	**10.9**	3 217 763	388 192 563	**8.3**
#3	7 063 941	539 404 010	**13.1**	1 224 927	230 604 186	**5.3**
#4	2 213 918	148 184 470	**14.9**	4 215 306	354 052 562	**11.9**
#5	4 027 597	378 109 299	**10.7**	1 974 487	210 307 731	**9.4**
#6	1 402 298	123 363 043	**11.4**	1 429 132	250 627 288	**5.7**
#7	2 498 967	182 824 193	**13.7**			
#8	2 105 343	208 395 146	**10.1**			
Total	26 612 397	2 141 574 070	**12.4**	13 896 733	1 626 884 301	**8.5**

B

	Intron 3' to JH4 spleen GC (Immunized)					
	wt mice			MARsEμ$^{\Delta/\Delta}$ mice		
# of individual mice	number of mutations	total number of bp analyzed	Frequency (mutation/Kb)	number of mutation	total number of bp analyzed	Frequency (mutation/Kb)
#1	8 799	1 416 237	**6.2**	5 535	3 257 438	**1.7**
#2	5 285	1 173 359	**4.5**	31 896	13 027 215	**2.5**
#3	59 657	19 245 534	**3.1**	482	501 374	**1.0**
Total	73 741	21 835 130	**3.4**	37 913	16 786 027	**2.3**

C

	Intron 3' to Imu Peyer's patches GC					
	wt mice			MARsEμ$^{\Delta/\Delta}$ mice		
# of individual mice	number of mutations	total number of bp analyzed	Frequency (mutation/Kb)	number of mutation	total number of bp analyzed	Frequency (mutation/Kb)
#1	48604	35 947 796	**1.4**	340 669	70 101 915	**4.9**
#2	42 937	32 953 275	**1.3**	1 098 398	215 265 958	**5.1**
#3	59 309	28 156 682	**2.1**	530 553	94 311 231	**5.6**
#4	17 594	11 681 044	**1.5**	241790	32530038	**7.4**
#5	11 901	3231298	**3.7**	192 502	23871828	**8.1**
#6	27 566	9864502	**2.8**	117 229	16868982	**6.9**
#7				343 232	44846733	**7.7**
Total	207 911	121 834 597	**1.7**	2 864 373	497 796 685	**5.8**

Supplementary Table S3

	# of unique rearrangement analyzed	**SHM in FR3** (downsream from VHJ558 consensus primer, defind by IMGT V-Quest)			**SHM in CDR3** (end of rearranged-VH segment, defined by IMGT V-Quest)			**SHM in FR3+CDR3**		
		# of nt analyzed	# of substitutions	SHM frequency (#mut/kbp)	# of nt analyzed	# of substitutions	SHM frequency (#mut/kbp)	# of nt analyzed	# of substitutions	SHM frequency (#mut/kbp)
Non-productive rearrangements to JH3 or JH4 in WT mice (n=3 samples)	*38*	*1438*	*36*	***25.03***	*187*	*14*	***74,87***	*1582*	*50*	***31.60***
Non-productive rearrangements to JH3 or JH4 in $MARS_{E\mu}$KO mice (n=2 samples)	*25*	*924*	*20*	***21.64***	*123*	*8*	***65.04***	*1047*	*28*	***26.74***

Supplementary Table S4

A

	Intron 3' to JH4 Peyer's patch GC					
	UNG $^{\Delta/\Delta}$ MSH2 $^{\Delta/\Delta}$ mice			UNG $^{\Delta/\Delta}$ MSH2 $^{\Delta/\Delta}$ MARsEμ $^{\Delta/\Delta}$ mice		
# of individual mice	number of mutations	total number of bp analyzed	Frequency (mutation/Kb)	number of mutation	total number of bp analyzed	Frequency (mutation/Kb)
#1	83 489	39 010 041	**2.1**	165 311	42 503 695	**3.9**
#2	542 601	70 241 171	**7.7**	94 241	16 931 788	**5.6**
#3	206 309	48 590 092	**4.2**	512 031	57 470 681	**8.9**
#4				1 041 060	149 775 567	**7**
#5				718 875	69 661 668	**10.3**
Total	832 399	157 841 304	**5.3**	2 531 518	336 343 399	**7.5**

B

	Intron 3' to JH4 Peyer's patch GC					
	UNG $^{\Delta/\Delta}$ mice			UNG $^{\Delta/\Delta}$ MARsEμ $^{\Delta/\Delta}$ mice		
# of individual mice	number of mutations	total number of bp analyzed	Frequency (mutation/Kb)	number of mutation	total number of bp analyzed	Frequency (mutation/Kb)
#1	272 454	26839331	**10.2**	613 978	89 213 320	**6.9**
#2	1 798 561	145965068	**12.3**	473 338	44 306 794	**10.7**
#3	432 270	160149949	**2.7**	441 712	67 366 607	**6.6**
#4	633 350	90533829	**7**	569 070	65 436 018	**8.7**
#5	507 031	47228276	**10.7**	817 177	73 685 315	**11.1**
#6	99 600	9066959	**10.9**	744 638	79 286 350	**9.4**
#7	208 684	19274286	**10.8**			
#8	258 678	34545602	**7.5**			
#9	318 557	30156482	**10.6**			
#10	1 576 407	163649903	**9.6**			
#11	2 822 217	195113384	**14.5**			
#12	563 458	56197886	**10**			
Total	9 491 267	978 720 955	**9.7**	3 659 913	419 294 404	**8.7**

C

	Intron 3' to Imu Peyer's patches GC					
	UNG $^{\Delta/\Delta}$ MSH2 $^{\Delta/\Delta}$ mice			UNG $^{\Delta/\Delta}$ MSH2 $^{\Delta/\Delta}$ MARsEμ $^{\Delta/\Delta}$ mice		
# of individual mice	number of mutations	total number of bp analyzed	**Frequency (mutation/Kb)**	number of mutation	total number of bp analyzed	Frequency (mutation/Kb)
#1	78 467	34 863 085	**2.3**	64 593	11 768 040	**5.5**
#2	117 529	19 114 688	**6.2**	794 845	81 456 430	**9.8**
#3	4 285	698 559	**6.1**	829 374	83 165 361	**10**
#4				394 268	34 269 808	**11.5**
Total	200 281	54 676 332	**3.7**	2 083 080	210 659 639	**9.9**

D

	Intron 3' to Imu Peyer's patches GC					
	UNG $^{\Delta/\Delta}$ mice			UNG $^{\Delta/\Delta}$ MARsEμ $^{\Delta/\Delta}$ mice		
# of individual mice	number of mutations	total number of bp analyzed	Frequency (mutation/Kb)	number of mutation	total number of bp analyzed	Frequency (mutation/Kb)
#1	154 039	46 862 890	**3.3**	566 494	78 527 619	**7.2**
#2	138624	45 368 381	**3**	944 483	141 666 058	**6.7**
#3	102 015	41 553 443	**2.5**	680 364	210 922 293	**3.2**
#4	155 773	63 882 794	**2.4**	91 352	11 480 275	**8**
#5	301 513	106 673 675	**2.8**	429 016	81 539 599	**5.3**
#6	431 266	144 210 474	**3**			
#7	468 727	144 538 082	**3.2**			
Total	1 751 957	593 089 739	**3**	2 711 709	524 135 844	**5.2**
